# Cancer‐Associated BCL‐2 Mutants Reveal Mechanisms Towards Venetoclax Resistance

**DOI:** 10.1002/advs.76693

**Published:** 2026-07-24

**Authors:** Jonas Aufdermauer, Ian de Ridder, Mahjoobeh Ehsani, Marasim Khan, Justin Kale, Lukas P. Frenzel, David Andrews, Spyridoula Karamanou, Ana J. Garcia‐Saez, Geert Bultynck

**Affiliations:** ^1^ Cologne Excellence Cluster on Cellular Stress Responses in Aging‐Associated Diseases University of Cologne Cologne Germany; ^2^ Department of Membrane Dynamics Max Planck Institute of Biophysics Frankfurt am Main Germany; ^3^ Department of Cellular & Molecular Medicine KU Leuven Leuven Belgium; ^4^ Department of Microbiology, Immunology, and Transplantation Rega Institute KU Leuven Leuven Belgium; ^5^ Department of Biological Sciences Sunnybrook Research Institute Toronto Canada; ^6^ Department of Internal Medicine University of Cologne Cologne Germany; ^7^ Center for Molecular Medicine Cologne University of Cologne Cologne Germany; ^8^ Department of Medical Biophysics University of Toronto Toronto Canada

**Keywords:** apoptosis, B‐cell lymphoma‐2, cancer, resistance mutations, venetoclax

## Abstract

BCL‐2 is an anti‐apoptotic protein frequently upregulated in cancer, enabling cell survival despite oncogenic stress. This BCL‐2 dependence sensitizes tumor cells to venetoclax, an FDA‐approved drug used against chronic lymphocytic leukemia. Yet, treatment‐induced mutations in BCL‐2 frequently result in resistance. While mutations G101V and D103Y were reported to disrupt BCL‐2 interaction with venetoclax, it remains unclear how other mutations cause resistance. Here, we performed a comprehensive comparison of frequently occurring cancer‐associated BCL‐2 mutations inside and outside the venetoclax binding site. We find that, besides disrupting venetoclax interaction, G101V and D103Y also increased the sequestration and inhibition of pro‐apoptotic proteins, revealing a double effect of these mutations. We also define V156D, A113G, R129L, and R139H as previously uncharacterized BCL‐2 mutations conferring venetoclax resistance. Remarkably, V156D reduces venetoclax binding allosterically, without altering pro‐apoptotic protein inhibition. Other BCL‐2 mutants, like A113G, R129L, and R139H, do not exhibit alterations in venetoclax binding, but venetoclax cannot efficiently release their pro‐apoptotic partners from inhibitory complexes in cells. Finally, sonrotoclax binds with high affinity to all venetoclax‐resistant BCL‐2 mutants, yet its efficacy against these BCL‐2 mutants in cells was heterogeneous. Our findings uncover diverse mechanisms that reduce venetoclax efficacy with the potential to inform anticancer treatment.

## Graphical abstract

Venetoclax (VEN) resistance in chronic lymphocytic leukemia arises from diverse BCL2 mutations. We map mechanisms contributing to VEN resistance across common BCL‐2 variants. G101V and D103Y reduce drug binding and increase sequestration of pro‐apoptotic proteins. V156D blocks VEN allosterically. A113G, R129L, and R139H allow binding yet prevent release at the mitochondria. These insights guide patient stratification and next‐generation BH3 mimetics such as sonrotoclax.

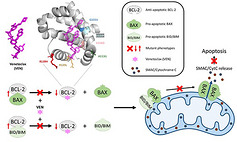

## 1 Introduction

The B‐cell lymphoma 2 (BCL‐2) family of proteins controls mitochondrial outer membrane permeabilization (MOMP), the process that determines the onset of apoptosis. MOMP enables the release of cytochrome c and SMAC/Diablo from the mitochondrial intermembrane space into the cytosol and the subsequent activation of caspases, leading to apoptosis [1]. Guardian BCL‐2‐protein family members such as BCL‐2, BCL‐XL, and MCL‐1 are anti‐apoptotic and bind and neutralize pro‐apoptotic members, including the executioners of MOMP such as BAX and BAK, as well as the initiator BH3‐only proteins. The BH3‐only proteins can either act as activators of BAX/BAK in the case of BID and BIM or as sensitizers by inhibiting anti‐apoptotic BCL‐2‐family members without directly activating BAX/BAK in the case of BAD and NOXA [2]. The sensitizer BH3‐only proteins interfere with BAX and BAK complexes with the guardians, so that they are released and can become activated. Structurally, guardian BCL‐2 proteins exert their anti‐apoptotic function by directly binding the surface‐exposed BH3 domain of pro‐apoptotic members into a hydrophobic cleft formed by their BH1‐3 domains, sequestering them into inactive complexes. However, other, less understood regions have also been implicated. This includes the BH4 domain of BCL‐2, which prevents BAX pore formation via a non‐canonical site in its N‐terminus [3], and a C‐terminal sequence in BIM responsible for binding BCL‐2, likely to a region outside the hydrophobic cleft [4].

Given their critical role in suppressing cell death, anti‐apoptotic BCL‐2 family proteins are overexpressed in a broad spectrum of malignancies, thereby enabling cancer cells to cope and survive with the upregulation of BH3‐only proteins that result from oncogenic stress [5]. In cancers, upregulated pro‐apoptotic proteins such as BAX/BAK and BIM are sequestered and kept in check by anti‐apoptotic BCL‐2 proteins. This also renders cancer cells highly vulnerable, placing them in an apoptosis‐primed state, as their survival becomes critically dependent on the presence and function of anti‐apoptotic BCL‐2‐family members [6]. This has led to the development of small‐molecule antagonists that mimic the BH3 domain of BH3‐only proteins. BH3 mimetics interfere with the anti‐apoptotic action of BCL‐2 family members by competing for binding to the hydrophobic groove, so that the pro‐apoptotic family proteins are released from the inhibitory complexes and free to oligomerize and induce apoptosis.

ABT‐199/venetoclax (in the following VEN) is a clinically used BH3 mimetic that selectively binds BCL‐2 and thus antagonizes its anti‐apoptotic activity without directly activating BAX/BAK [7]. VEN displays a high efficacy in treating hematologic malignancies typified by high BCL‐2‐protein levels, such as chronic lymphocytic leukemia (CLL) and small lymphocytic lymphoma, and acute myeloid leukemia, especially in combination with other therapeutics such as BCR inhibitors or monoclonal antibodies [8, 9, 10, 11]. Despite this important progress, long‐term treatment of CLL with VEN has brought up new challenges [7]. Many CLL patients, especially in later stages of disease treatment, relapse and become resistant to VEN [12, 13, 14, 15]. Multiple mechanisms for VEN resistance have been proposed, including upregulation of alternative anti‐apoptotic proteins, such as MCL‐1 and BCL‐XL [16, 17, 18, 19, 20], loss‐of‐function mutations in BAX [21], overexpression of RNA methylation proteins [22], hyperphosphorylation of BCL‐2 family members, such as MCL‐1, BAD and BAX [23], and finally also mutations in BCL‐2 itself that alter VEN binding without abrogating its ability to bind pro‐apoptotic proteins, thereby retaining proper anti‐apoptotic function [24, 25]. The most frequently reported acquired VEN‐resistance mutations in BCL‐2 include G101V and D103Y, which were identified in relapsed CLL patients and shown to reduce VEN binding by sterically interfering with the BH3‐binding groove [24, 26, 27, 28]. In a targeted ultra‐deep sequencing study of 67 CLL patients, G101V and D103Y are each observed in ∼10% of relapsed CLL patients, with 6% harboring both mutations [29]. Strikingly, almost all patients harboring the G101V and/or D103Y mutation experienced relapse and showed clear signs of disease progression. Others report that about one third of patients on long‐term VEN treatment obtained the G101V mutation, often emerging at low frequency and increasing upon disease progression [30]. In addition to G101V and D103Y, other BCL‐2 mutations have been identified in CLL patients experiencing disease progression during prolonged VEN treatment, namely A113G, R129L, and V156D [28, 29, 31, 32, 33]. One study on relapsed CLL patients pretreated with VEN showed that such additional recurrent mutations occur in 91% of patients in whom the G101V mutations were previously identified [32]. An average of three mutations was detected in each patient, with large proportions of the CLL compartments in these patients containing recurrent mutations. However, the mutations A113G, R129L, and V156D individually account for only a small portion of 1%–3% of VEN‐treated CLL patients, together amounting to about 10% of BCL‐2 mutant cases. While resistance mutations might occur concurrently, they were reported to be present in different reads of the NGS data, consistent with their prevalence in different cells and thereby assuming heterozygosity [32].

Interestingly, several BCL‐2 mutations have also been identified outside the context of VEN treatment, with mutations R6K, R12W, A113G, R129L, R139H, and V156D having been observed in either diffuse large B‐cell lymphoma (DLBCL) [34], follicular lymphoma (FL), or both [35]. In FL, R6K, A113G, R139H, and V156D were associated with increased disease progression [35]. Mutation A4T was identified in lymphoma cells after prolonged exposure to gradually increasing VEN concentrations [36]. As these cancer‐related BCL‐2 mutations occur in different BCL‐2 domains implicated or not in BAX and/or BH3‐only protein binding, it is critical to understand how these mutations impact the anti‐apoptotic function of BCL‐2, particularly BAX inhibition, as well as the efficacy of VEN.

With this in mind, we set out an integrative set of biochemical, biophysical, and cell biological approaches to study the impact of BCL‐2 mutations related to cancer on VEN resistance. Our findings indicate that all cancer‐associated BCL‐2 mutants continue to bind BH3‐only proteins and BAX. These BCL‐2 mutants also retain the ability to prevent BAX‐induced MOMP and subsequent cell death. However, only mutations G101V, D103Y, R129L, and V156D render the BCL‐2 protein profoundly more resistant to exposure to VEN, in line with hindered VEN‐induced disruption of BCL‐2 interactions with the BH3 domain of BIM and with BAX in vitro and in cells. This results in reduced de‐repression of BAX‐mediated pore formation and cell death. These insights provide a framework by which cancer‐associated mutations in BCL‐2 can, on one hand, impact BCL‐2's biological function in cells and, on the other hand, affect BCL‐2's pharmacological targeting by VEN. Ultimately, our findings hold promise to inform the design and analysis of next‐generation BH3‐mimetic BCL‐2 inhibitors and improve therapeutic strategies for patients with lymphoma with BCL‐2 mutations.

## 2 Results

### 2.1 VEN Ability to Displace BH3^BIM^ From BCL‐2 Cancer‐Related Mutants Does not Correlate With BCL‐2/BH3^BIM^ Affinity

**FIGURE 1:**
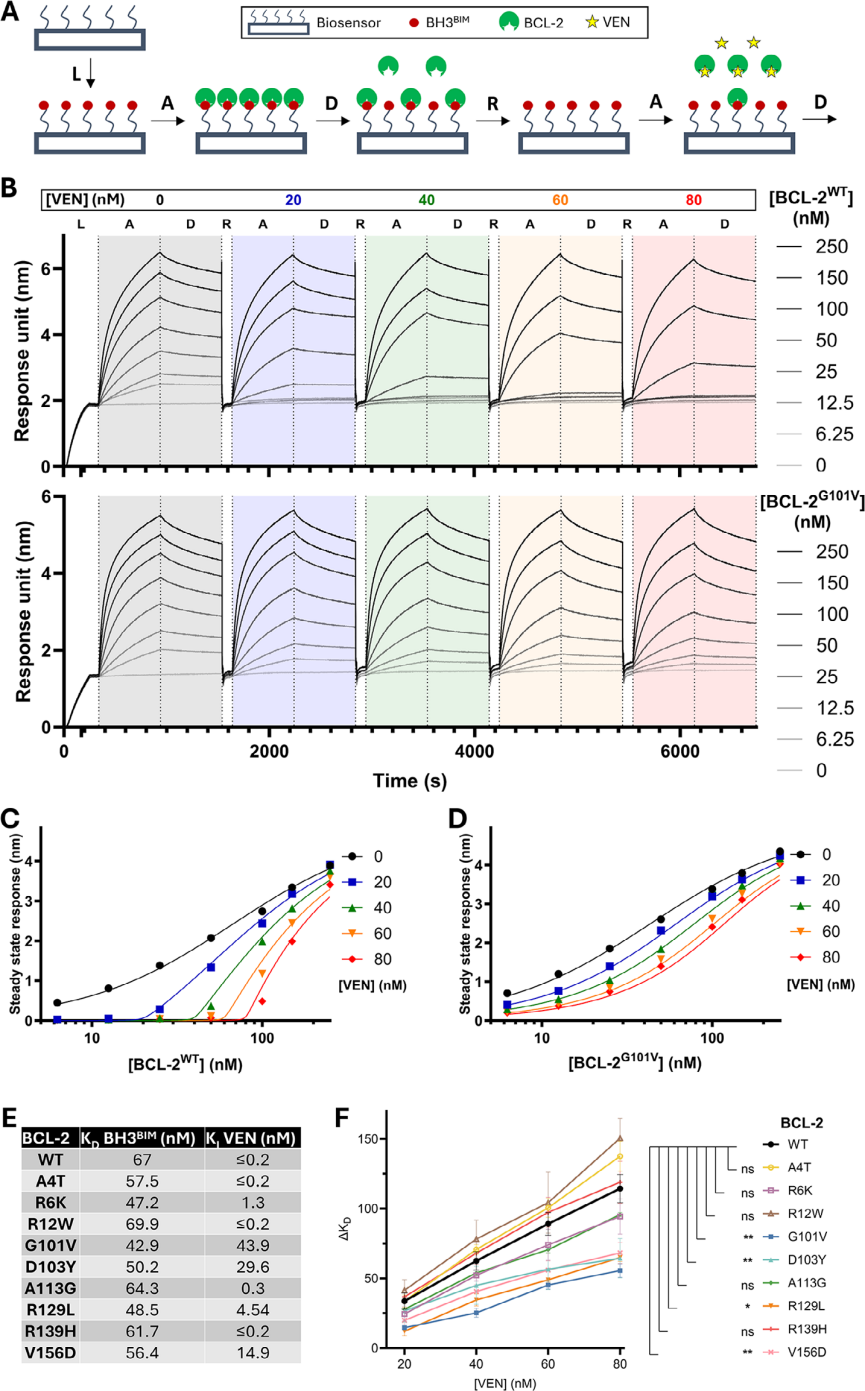
Effect of BCL‐2 mutations on venetoclax (VEN)‐induced displacement of a BIM peptide. (A) A representative model of the performed competitive, indirect interaction analysis through BLI. Biosensors were loaded with BH3^BIM^ peptides in the loading phase (“L”). Binding affinities of purified BCL‐2^∆TMD^ proteins to immobilized BH3^BIM^ were determined in the association phase (“A”) and dissociation phase (“D”). Afterward, the biosensors were regenerated (“R”) and reused to determine the affinity of BH3^BIM^ toward the same BCL‐2^∆TMD^ protein in the presence of the indicated VEN concentration. This was repeated for all VEN concentrations. (B) Exemplary BLI traces of the evaluation of one biological wild‐type BCL‐2^∆TMD^ (top) and BCL‐2^∆TMD^ G101V (bottom) replicate, respectively. (C, D) Binding curves illustrate the interactions between BH3^BIM^ and wild‐type BCL‐2^∆TMD^ (C) and BCL‐2^∆TMD^ G101V (D), respectively, incubated with various VEN concentrations (different colors), which are displayed as average steady‐state responses determined at the end of each association phase (595‐600 s). The steady‐state values were fitted using Equation (2) to obtain K_D_ for the interaction between BH3^BIM^ and BCL‐2^∆TMD^ and K_I_ values for VEN. Each data point reflects the mean ± S.D. for *n* = 3. (E) Table showing the results of the performed BLI experiments evaluating the BCL‐2^∆TMD^ protein interaction with BH3^BIM^ and VEN in K_D_ or K_I_ (averaged). (F) VEN‐induced shifts in BH3^BIM^‐BCL‐2^∆TMD^ binding kinetics are plotted, showing the differential K_D_ values at each VEN concentration in relation to the control condition.

We first assessed the impact of the selected cancer‐related BCL‐2 mutations on their binding affinity to BH3^BIM^, a synthetic peptide containing the sequence of the BH3 domain of BIM, as well as on the potency of VEN to disrupt the BCL‐2/BH3^BIM^ interaction in solution. For this, we performed an in vitro interaction assay based on biolayer interferometry (BLI), using different concentrations of purified wild‐type and mutant BCL‐2 proteins and of BH3^BIM^ and increasing concentrations of VEN. The BH3^BIM^ peptide was immobilized to the streptavidin‐coated sensor as a biotinylated peptide. We note that all recombinantly purified BCL‐2 proteins used in our assays lack their C‐terminal transmembrane domain (TMD) to ensure robust BCL‐2 purification and behavior in solution. A schematic view of the BLI principle and our experimental setup is presented in Figure 1A. Representative raw BLI data of one biological replicate are shown in Figure 1B, and the corresponding fitting of the data for BCL‐2^∆TMD^ vs. BCL‐2^∆TMD^‐G101V are depicted in Figure 1C,D, respectively. The analysis of BCL‐2^∆TMD^ G101V serves as validation of our setup, as this mutation has already been shown to reduce VEN‐induced disruption of BCL‐2/BH3^BIM^ 180‐fold when compared to wild‐type in a very similar surface plasmon resonance assay [24].

After immobilizing the BH3^BIM^ peptide (see “L” in Figure 1B), we exposed the sensor to different concentrations of BCL‐2^∆TMD^ or BCL‐2^∆TMD^‐G101V proteins and tracked their association for 600 s (see “A” in Figure 1B). We then induced dissociation of the bound proteins for 600 s by exposing them to the binding buffer alone (see “D” in Figure 1B). Next, the sensor was briefly regenerated (10 s), and the exact same experiment was repeated in the presence of increasing VEN concentrations (20, 40, 60, and 80 nM). Of note, the association curves with the highest BCL‐2^∆TMD^ concentration reached similar values for each condition, indicating that the regeneration did not affect the overall BCL‐2‐binding properties of the immobilized BH3^BIM^ peptide. We calculated the dissociation constant (K_D_) values for BCL‐2 binding to BH3^BIM^ by fitting the steady‐state data, yielding a high‐affinity binding K_D_ of ∼67 nM for BCL‐2^∆TMD^. The fittings of the data obtained for the remaining BCL‐2^∆TMD^ mutants are presented in Figure S1. None of the mutations caused a striking change in K_D_ for binding to BH3^BIM^. Most mutants displayed a somewhat lower K_D_ value (Figure 1E), indicating that the mutations do not perturb binding to BH3 domains of pro‐apoptotic proteins and may slightly improve it, thus potentially mildly enhancing their anti‐apoptotic function.

We also determined the inhibitory constant (K_I_) values for dissociation of the BCL‐2^∆TMD^/BH3^BIM^ complex by VEN in line with Birkinshaw et al. [27]. For the K_I_ value of BCL‐2^∆TMD^ and BH3^BIM^, we set the cut‐off at 0.2 nM, as the lowest value that could be accurately determined. Remarkably, we found that not only G101V and D103Y, but also R129L and V156D displayed K_I_ values that were at least ∼20‐fold higher. In contrast, the K_I_ values for R6K and A113G were slightly higher, while A4T, R12W, and R139H behaved similarly to wild‐type BCL‐2^∆TMD^. To strengthen this analysis, we also estimated the shift in K_D_ (∆K_D_) for BCL‐2^∆TMD^ binding to BH3^BIM^ in the presence of increasing VEN concentrations (Figure 1F). As expected, due to VEN competition with BH3^BIM^ for binding to BCL‐2^∆TMD^, the apparent K_D_ for BCL‐2^∆TMD^ binding to BIM increased with increasing VEN concentration. In case that a mutation would render the BCL‐2 mutant more resistant to competition with VEN for binding to BH3^BIM^, then the ∆K_D_ would be lower than for BCL‐2^∆TMD^; and vice versa for mutations causing increased susceptibility to competition with VEN. Interestingly, G101V, D103Y, R129L, and V156D displayed significantly lower ∆K_D_ values than those obtained for BCL‐2^∆TMD^, confirming that these mutants are less susceptible to VEN competition for BH3^BIM^. BCL‐2^∆TMD^ R6K and A113G displayed a tendency to lower ΔK_D_ values compared to BCL‐2^∆TMD^, suggesting a minor resistance effect. Instead, BCL‐2^∆TMD^ A4T, R12W, and R139H displayed similar ΔK_D_ values as wild‐type, with A4T and R12W being slightly more sensitive.

### 2.2 BCL‐2 Mutations G101V, D103Y, R129L, R139H, and V156D Reduce Binding to VEN

**FIGURE 2:**
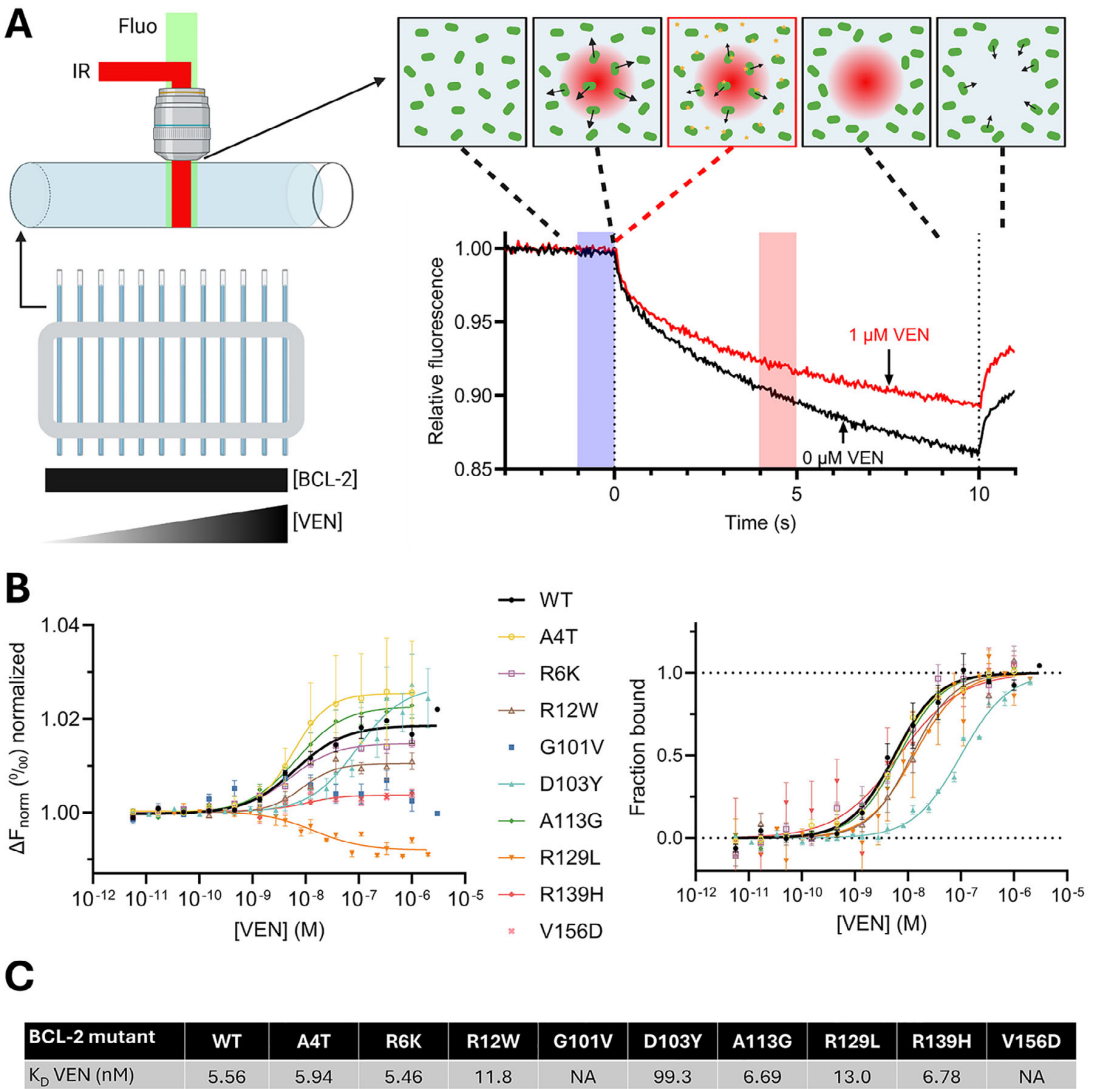
Direct binding of venetoclax (VEN) to different BCL‐2 mutants. (A) A representative model of the performed direct interaction analysis through MST. An infrared (IR) laser heats a tiny spot in the sample within a capillary, creating a temperature gradient. Molecules move (thermophoresis) depending on their size, charge, and hydration, which all change when they bind. Fluorescence (Fluo) is measured to track this movement, and any changes between samples reveal binding interactions. Measured fluorescence of RED‐labeled purified 6xHis‐tagged BCL‐2^∆TMD^ proteins (10 nM) in the presence (red) and absence (black) of a 1 µM VEN is represented as an MST trace experiment. Within the graph, the blue shaded (cold) area signifies the data point used for normalization of mean fluorescence, while the red shaded (hot) area indicates the data points used for the quantification of the thermophoretic shift as a function of VEN concentration. The dotted vertical lines indicate the time points when the infrared laser was activated (right) and deactivated (left). (B) Right: Binding curves displaying observed thermophoretic shift in function of VEN concentration. The fitted curves illustrate the interaction between purified and RED‐labeled 6xHis‐tagged BCL‐2^∆TMD^ (10 nM) and VEN. The y‐axis (ΔF_norm_) represents the ratio of normalized fluorescence (F_norm_), which is again normalized using the ΔF_norm_ corresponding to the lowest respective BCL‐2^∆TMD^ protein concentration. The x‐axis depicts a logarithmic scale of the corresponding VEN concentration. Each data point reflects the mean ± SEM for *n* = 3 biological measurements, with each between 1 and 3 technical replicates. Left: corresponding analyzed data. The y‐axis (fraction bound) represents the normalized fluorescence (F_norm_), which is transformed using the plateau phases of each binding curve. (C) Table showing the results of the performed MST experiments evaluating the interaction of BCL‐2^∆TMD^ with VEN in K_D_. NA: data not available or not applicable for this condition.

As our previous dataset indicated a different susceptibility of the BCL‐2^∆TMD^/BH3^BIM^ complex to dissociation by VEN, we wondered whether this was due to changes in VEN affinity for BCL‐2. We therefore examined the direct interaction between VEN and recombinant BCL‐2^∆TMD^, wild‐type or mutant, using microscale thermophoresis (MST). MST enables sensitive detection of binding‐induced changes in the hydrodynamic radius of fluorescent particles by detecting shifts in their thermophoretic mobility and is therefore well‐suited for studying direct interactions between a protein and small molecules in solutions (schematic representation of our experimental setup in Figure 2A; Figure S2). Fluorescently labelled wild‐type and mutant BCL‐2^∆TMD^ proteins were titrated with increasing concentrations of VEN up to 1 µM to determine their binding affinities. The shift in thermophoretic mobility was normalized and plotted as ∆F_norm_, whereby the thermophoretic mobility of BCL‐2^∆TMD^ at baseline was set at 1. The average thermophoretic mobility between 4 and 5 s was used for further analysis. To determine the K_D_, we calculated the fraction of BCL‐2^∆TMD^ bound to VEN as a function of the VEN concentration for the curves displaying a clear concentration‐dependent profile.

Wild‐type BCL‐2^∆TMD^ bound VEN with high affinity, with a K_D_ of approximately 6 nM (Figure 2B,C), consistent with previously reported values [24, 26]. We also determined the VEN binding to all BCL‐2^∆TMD^ mutants. Importantly, BCL‐2^∆TMD^ G101V and V156D did not display a clear concentration‐response curve. This indicates that either the affinity of the BCL‐2^∆TMD^ mutant to VEN is below the detection limit or that the binding of VEN to the BCL‐2^∆TMD^ mutant does not induce a strong enough change in the hydrodynamic radius of these mutants. For all other mutants, a concentration‐response curve could be determined, despite the small thermal shift induced by VEN. Although the level of displacement was very low for R139H, the curve displayed a concentration‐dependent profile. We found that the K_D_ of D103Y (∼100 nM, being a ∼20‐fold reduction in affinity) and R129L (∼13 nM, being a ∼3‐fold reduction in affinity) were increased compared to wild‐type BCL‐2^∆TMD^ (∼6 nM). The direct binding of VEN to BCL‐2^∆TMD^ was only mildly or not affected by A4T, R6K, R12W, A113G, and R139H mutations. These results mirror the impaired VEN displacement experiments for G101V, D103Y, R129L, and V156D observed via BLI. Overall, G101V, D103Y, and V156D appear to cause the most dramatic decrease in VEN binding of BCL‐2^∆TMD^, while mutation R129L only results in a minor decrease in VEN‐binding affinity.

### 2.3 Structural and Dynamic Shifts in the VEN‐Binding Site of Disease‐Associated BCL‐2 Mutants

**FIGURE 3:**
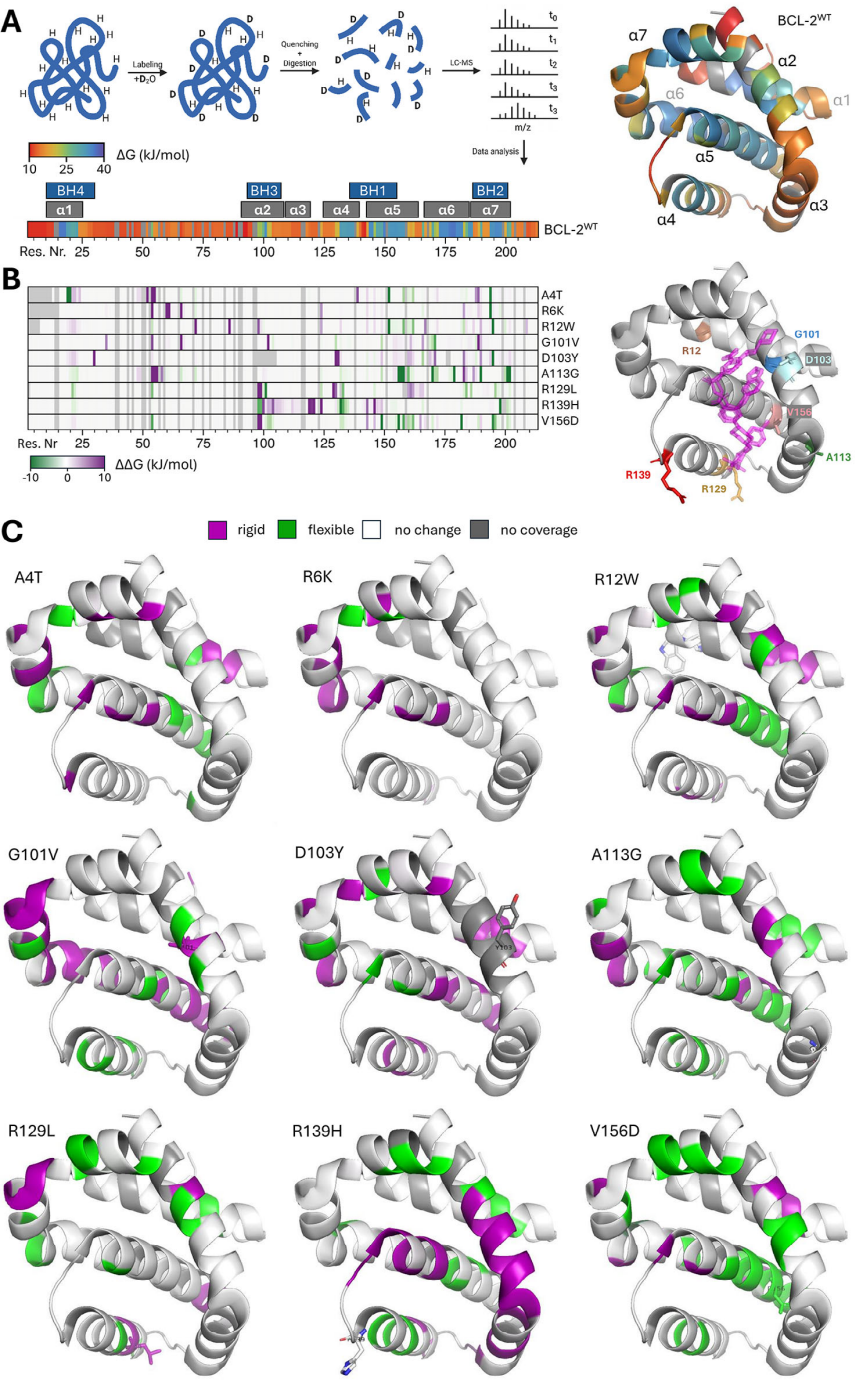
Changes in BCL‐2 structural dynamics by cancer‐related mutations. (A) Schematic overview of the hydrogen‐deuterium exchange‐mass spectrometry (HDX‐MS) process to acquire Gibbs free energy values (ΔG) for the residues of the recombinant BCL‐2^∆TMD^ protein. A linear representation of the ΔG of wild‐type recombinant BCL‐2^∆TMD^ is shown below with annotation of the functional domains (blue) and the secondary structures (grey) within the BCL‐2^∆TMD^ protein above. We mapped the ΔG data on the crystal structure of the wild‐type human BCL‐2 protein (P10415), of which a 2D representation is shown on the right. (B) Linear representation of differences in ΔG of recombinant BCL‐2^∆TMD^ mutants to wild‐type, colored according to the per‐residue value in ΔΔG. The respective mutations in each of the mutants are shown to the right of each linear representation. Regions in purple are rigidifying when compared to the said region in wild‐type BCL‐2^∆TMD,^ while regions in green loosen. Right: 2D representation of the 3D protein structure of wild‐type BCL‐2^∆TMD^ with VEN (VEN) (magenta, see‐through) docked within its binding site. The mutated residues of interest in the BCL‐2^∆TMD^ are highlighted in different colors. Amino acids 1–9, as well as other flexible regions, are missing from the protein's crystal structure (P10415). As such, the mutations within this region, as well as any results corresponding to these residues, are not displayed in any of the shown protein structures. (C) The ΔΔG of the evaluated recombinant BCL‐2^∆TMD^ mutants relative to wild‐type BCL‐2^∆TMD^ mapped on the protein structure of wild‐type BCL‐2^∆TMD^ (P10415). The respective mutations are presented above each structure. Regions in purple show significantly increased ΔΔG (rigid), which corresponds with decreased accessibility of the hydrogen atoms of this residue with deuterium relative to wild‐type BCL‐2^∆TMD^, while regions in green represent significantly decreased ΔΔG (flexible). Residues for which no reliable data could be obtained are shown in dark grey.

To gain more insight into the impact of the disease‐associated mutations on the structural dynamics of BCL‐2^∆TMD^ in solution, we performed hydrogen–deuterium exchange mass spectrometry (HDX‐MS) on recombinant BCL‐2^∆TMD^ and the cancer‐related mutants of BCL‐2^∆TMD^. This approach allows for a residue‐level evaluation of exchange rates of hydrogen to deuterium, providing information about the local environments in terms of surface exposure and rigidity, yielding ΔG values as schematically represented in Figure 3A. Although the measurements with BCL‐2^∆TMD^ derived mutants in solution may not fully recapitulate the full‐length protein in the membrane, we reasoned that our simplified system could still provide valuable insights into the structural effects of cancer‐related mutations on BCL‐2. This analysis indicated that generally BCL‐2^∆TMD^ (Figure 3A) has low ΔG values of 10–15 kJ/mol, indicating high H → D exchange rates and thus, high surface exposure. However, the amino acid regions spanning residues 15–25 corresponding to the BH4 domain, residues 95–105 corresponding to the BH3 domain, and residues 130–204 containing the BH1 and BH2 domains display high ΔG values of 25 kJ/mol and higher, indicating lower H → D exchange rates and thus limited surface exposure. Next, we determined the ΔG profile of the different BCL‐2^∆TMD^ mutants (Figure S3A) and calculated the differences with the wild‐type, analyzed as ΔΔG, by using the ΔG values (HDX‐MS profile) of BCL‐2^∆TMD^ as a reference (Figure 3B). In order to facilitate visual evaluation of our results and their structural implications, we mapped the obtained ΔΔG values of each mutant onto the homology model of the BCL‐2^∆TMD^ protein (Figure 3C, P10415). This analysis allows us to identify which regions in the BCL‐2 protein structure are particularly affected by the cancer‐related mutations.

We found that each mutation causes a distinct shift in ΔG compared to BCL‐2^∆TMD^, thus displaying a unique ΔΔG fingerprint with changes in different regions of the proteins. We observed both decreasing and increasing in the H → D exchange in all BCL‐2^∆TMD^ mutants among different parts of the protein relative to BCL‐2^∆TMD^. Moreover, several mutations caused long‐range, allosteric changes in deuterium accessibility in regions distant from their location, both when considering the primary sequence as well as the tertiary structure. These allosteric effects suggest that BCL‐2 is a highly dynamic protein in which local perturbations can variably impact overall conformational flexibility and solvent accessibility at remote sites. Several of the BCL‐2 mutants studied altered BCL‐2^∆TMD^ protein dynamics in residues directly involved in VEN binding. For instance, the G101V mutant provoked high shifts in the deuterium accessibility in the α‐helical BH3 domain (residues 95–105) (Figure 3B,C) [24, 26, 27, 31, 32, 34]. This shift is structurally adjacent to the site within the VEN‐binding pocket and is believed to play a crucial role in the resistance mechanisms of G101V [27, 37]. Similarly, we observed high structural shifts in the α‐helix corresponding to the BH3 domain of BCL‐2^∆TMD^ mutants R129L, R139H, and V156D, with both increased and decreased deuterium uptake when compared to wild‐type BCL‐2^∆TMD^ (Figure 3B,C). For the V156D, we observed that the aryl‐Cl group of VEN is theoretically spaced 4.7 Å from the V156 residue. The shift toward the negatively charged polar aspartic acid residue might directly affect binding through altered van der Waals contacts and/or electrostatic or polarization within the hydrophobic pocket. Unfortunately, for D103Y, due to poor resolution in the region carrying the mutation and strict analysis criteria, we were unable to resolve the ΔG for the residues in this area. Only two peptides covered this region, each containing the D103Y mutation (Figure S3B), which differ from the peptides for the wild‐type BCL‐2, rendering our analysis unable to determine ΔG and ΔΔG values for the covered residues. In contrast to the mutants with mutations located in the hydrophobic cleft, BCL‐2^∆TMD^ mutants A4T, R6K, and R12W displayed limited shifts in residues within the VEN‐binding pocket.

We also performed circular dichroism (CD) spectral analysis on recombinant wild‐type and mutant BCL‐2^∆TMD^ proteins to evaluate potential structural perturbations that could influence protein folding or stability. The melting temperature (T_m_) of BCL‐2^∆TMD^ (studied at 10 µM) wild‐type and the mutants were very similar, with the largest shift in T_m_ observed for BCL‐2^∆TMD^ V156D (T_m_ of 54.6 °C compared to 51.3 °C for wild‐type BCL‐2^∆TMD^) (Table 1). In the presence of 20 µM VEN, all BCL‐2^∆TMD^ proteins displayed a significantly increased T_m_. However, mutations G101V, D103Y, R139H, and V156D display a lower VEN‐induced increase in T_m_ when compared to wild‐type BCL‐2^∆TMD^, in line with the reduced binding of VEN to these mutants. Conversely, mutant R129L shows an increased VEN‐induced T_m_ shift.

**TABLE 1:** The average melting temperatures (T_m_) extracted from the CD‐spectral analyses performed on 10 µM recombinant BCL‐2^∆TMD^ protein in the absence (T_m_^Apo^) and presence (T_m_^Holo^) of 20 µM VEN. The difference in T_m_ of each BCL‐2^∆TMD^ mutant between its Apo and Holo states is also depicted.

Protein	T_m_^Apo^ (°C)	T_m_^Holo^ (°C)	Difference (T_m_^Holo^−T_m_^Apo^)
BCL‐2^WT^	51.25	70.75	19.51
BCL‐2^A4T^	50.06	71.56	21.22
BCL‐2^R6K^	53.27	72.78	19.51
BCL‐2^R12W^	50.81	70.81	20.00
BCL‐2^G101V^	53.00	67.25	14.25
BCL‐2^D103Y^	56.57	67.07	10.50
BCL‐2^A113G^	50.06	69.52	19.46
BCL‐2^R129L^	53.31	79.06	25.75
BCL‐2^R139H^	50.51	65.51	15.00
BCL‐2^V156D^	54.56	70.32	15.75

### 2.4 BCL‐2 Mutations G101V, D103Y, and R129L, R139H Compromise the VEN‐Induced Retrotranslocation of tBID From Mitochondria

**FIGURE 4:**
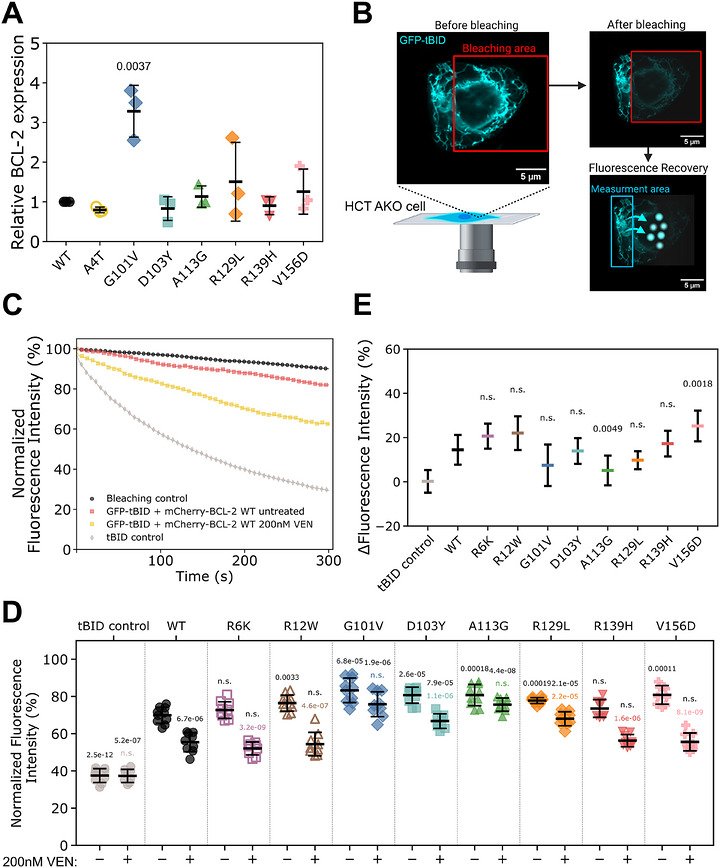
Impact of BCL‐2 mutations on its ability to retrotranslocate tBID from mitochondria. HCT AKO cells were co‐transfected with either GFP‐tBID alone or together with mCherry‐BCL‐2 wild‐type or mutated as indicated. (A) Quantification of mCherry‐BCL‐2 expression western blot in HCT AKO cells (Figure S4A) with average values and standard deviations (*n* = 3). For the statistical analysis, an independent t‐test comparison between wild‐type and mutant was performed. (B) Schematic experimental setup with confocal image of mitochondrial GFP‐tBID (cyan). The red box indicates the bleached area, and the blue box indicates the measurement area for FLIP. (C) Corresponding bleaching curves with mCherry‐BCL‐2 wild‐type untreated in purple, mCherry‐BCL‐2 wild‐type treated with 200 nM VEN for 24 h in yellow, and GFP‐tBID only control in brown. All intensity measurements were normalized to time point zero. (D) Evaluation of normalized fluorescence intensity loss at 300 s for all tested BCL‐2 mutants under untreated (‐) and treated with 200 nM VEN for 24 h (+) conditions. For statistical analysis, unpaired two‐tailed Welch's t‐tests with Holm–Bonferroni correction were used to compare wild‐type and mutant under untreated and 200 nM VEN‐treated conditions (black). Within each genotype, comparisons between untreated and 200 nM VEN‐treated cells are shown and color‐coded by sample. For each sample, *n* = 8–10 cells were evaluated on at least 3 different days. (E) Differences in normalized fluorescence intensity loss after 300 s untreated minus treated with 200 nM VEN for 24 h. For statistical analysis, a two‐way ANOVA (factors: genotype and treatment, Holm–Bonferroni corrected) was used to assess the combined effects of genotype and treatment and their interaction.

Next, we examined the impact of the most interesting BCL‐2 mutations in a cellular context. For this, we selected G101V, D103Y, A113G, R129L, R139H, and V156D because of their reduction in VEN binding and the changes in deuterium exchange for these BCL‐2 mutants. We first transiently overexpressed mCherry‐tagged versions of wild‐type and mutant BCL‐2 in HCT‐116 cells lacking most BCL‐2‐family proteins (in the following, HCT AKO) [38] and analyzed their expression levels by western blot analysis, which were largely comparable (Figure 4A; Figure S4A). We observed that G101V displayed significantly higher protein levels compared to the wild‐type BCL‐2 (Figure 4A). For comparison, we optimized transfection conditions to achieve similar mCherry fluorescence across wild‐type and mutant BCL‐2 construct G101V, allowing analysis at matched protein abundance (Figure S4B). While protein overexpression and the mCherry tag might introduce differences to the untagged, endogenous BCL‐2, these systems complemented our in vitro approaches by allowing us to study the effect of mutations in the full‐length protein and in the more physiological context of the cellular membranes.

BCL‐2 not only prevents apoptosis by sequestering pro‐apoptotic BCL‐2 proteins but also by retrotranslocating BAX and BAK from the mitochondria to the cytosol [39]. We previously showed that while BCL‐XL promotes BAX retrotranslocation, it enhances the accumulation of tBID at mitochondria in inhibitory complexes [40]. Building on this, we used Fluorescence Loss In Photobleaching (FLIP) to study the effect of cancer‐related mutations in BCL‐2 on tBID retrotranslocation. In these measurements, we estimated the ability of tBID to shuttle between the mitochondria and other cytoplasmic compartments in HCT AKO cells expressing GFP‐tBID either alone or together with mCherry‐BCL‐2. The lack of other BCL‐2 family proteins in this experimental system allowed us to disentangle the effect of BCL‐2 on tBID subcellular dynamics from other BCL‐2‐family members.

We bleached an area comprising approximately 80 % of the cell (Figure 4B, red square) and measured the fluorescence intensity in the area corresponding to the remaining unbleached 20 % of the cell. The fluorescence intensity loss caused by unbleached molecules shuttling into the cytosol and diffusing toward the bleached area provides information about protein dynamics. A control measurement on a nearby unbleached cell with similar intensity was performed under the same conditions to correct for potential photobleaching associated with imaging (Figure 4C). In the absence of BCL‐2, tBID presented a high exchange rate between mitochondria and cytosol, indicated by the fast loss of fluorescence during the FLIP measurements (Figure 4C). When expressed together with wild‐type BCL‐2, the exchange rate was significantly reduced, indicating BCL‐2 stabilizes tBID at mitochondria and reduces its ability to retrotranslocate (Figure 4D), in line with our previous observations for BCL‐XL [40]. This mitochondria‐stabilizing effect of wild‐type BCL‐2 on tBID was counteracted by the addition of 200 nM VEN (Figure 4D).

Interestingly, BCL‐2 G101V, D103Y, A113G, R129L, and V156D displayed a tendency for enhanced tBID stabilization on mitochondria in untreated cells, with G101V having the most prominent effects (Figure 4D). Furthermore, G101V, D103Y, A113G, and R129L appeared more resistant to the VEN‐mediated increase in tBID retrotranslocation in the presence of BCL‐2 (Figure 4D). In contrast, R6K, R12W, R139H, and V156D mutants, while still reducing tBID shuttling, remained susceptible to VEN‐induced changes in tBID retrotranslocation (Figure 4D). The differences between the normalized fluorescence intensity of untreated minus treated with 200 nM VEN are shown in Figure 4E. This analysis reveals that VEN has a significantly different effect due to the genotype only for A113G and V156D. For the others, this effect is dampened because they already have elevated normalized fluorescence intensity in untreated conditions, such that the shift at the end is similar to wild‐type BCL‐2. In summary, these results indicate that BCL‐2 reduces the retrotranslocation of tBID from mitochondria to the cytosol in a way that can be modulated by VEN, and that cancer‐related mutations in BCL‐2 can impact both their subcellular dynamics and their susceptibility to VEN.

### 2.5 Mutations in BCL‐2 Associated With Lymphoma Change its Binding Properties to BAX and tBID in the Mitochondria of Cells and Reduce Complex Displacement by VEN

To compare the interaction of wild‐type vs. cancer‐related mutants of BCL‐2 with tBID directly in the mitochondria of living cells, we used fluorescence cross‐correlation spectroscopy (FCCS). FCCS is a powerful tool to quantify diffusion coefficients, local concentration, and complex formation of individual proteins tagged with distinct fluorophores in cells. Single‐molecule‐level intensity fluctuations of the labeled proteins of interest are detected in the corresponding detection channels of a confocal microscope volume, and the cross‐correlation of the signal provides a quantitative analysis of complex formation directly in the context of the cell. The scanning version of this method has been used to study interactions between BCL‐2 proteins in vitro, and the extension to cells allows quantification of protein‐protein interactions directly in mitochondria [41, 42, 43].

**FIGURE 5:**
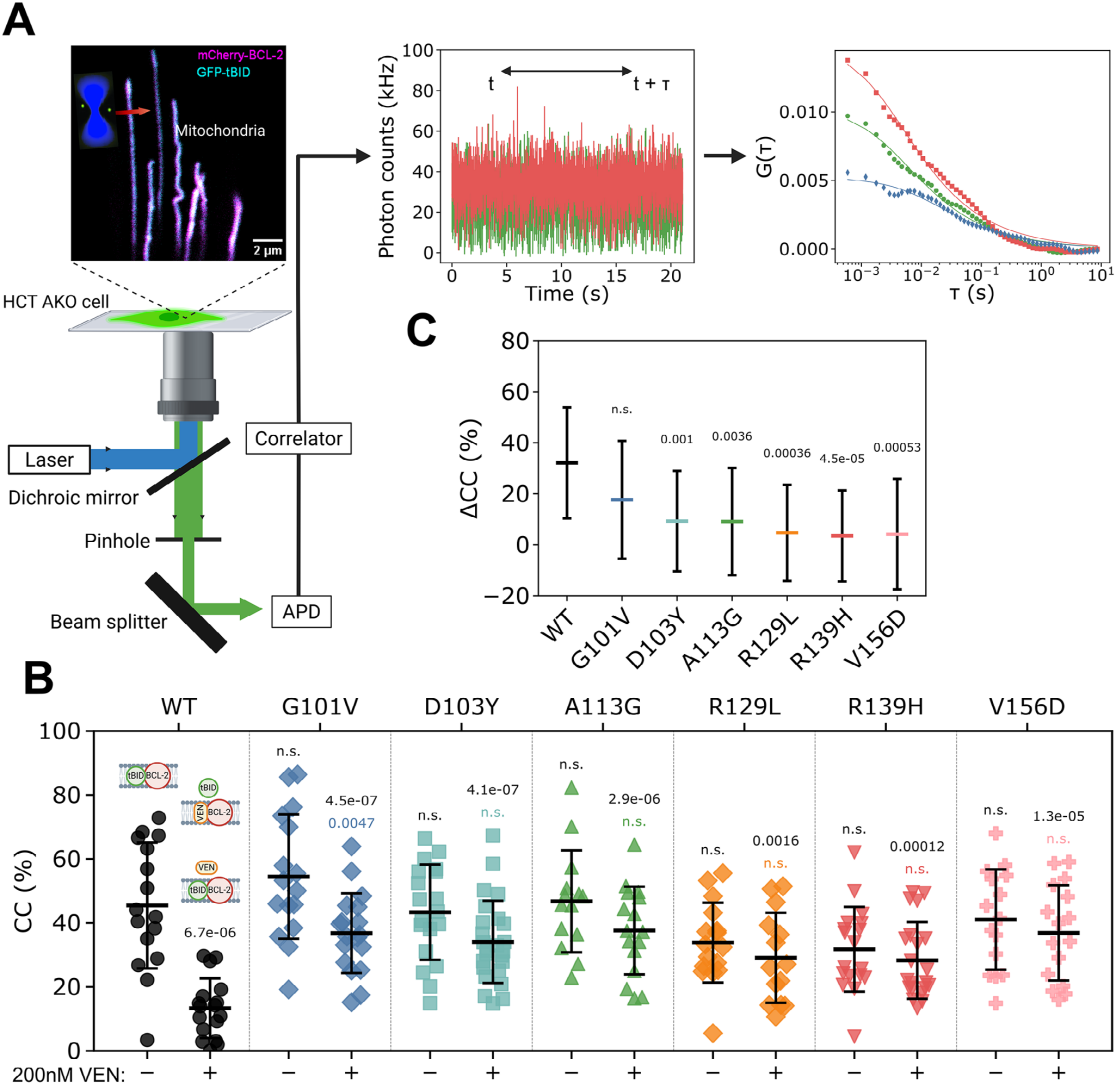
Venetoclax (VEN) cannot disrupt the interaction of tBID with BCL‐2 cancer‐related mutants in cells. HCT AKO cells were co‐transfected with either GFP‐BAX or GFP‐tBID and mCherry‐BCL‐2 wild‐type or mutated as indicated. (A) Schematic setup of SFCCS in mitochondria of living HCT AKO cells. 2 lasers of different colors (488 nm and 561 nm) are scanned through the region of interest depicted in the confocal image containing mCherry‐BCL‐2 (cyan) and GFP‐tBID (magenta). The red arrow indicates the line scanning path of the SFCCS. The signal is then collected by an Avalanche Photodiode (APD) and correlated, yielding an intensity fluctuation trace. For each timepoint t of the intensity trace, diffusion curves as a function of the lag time τ are then calculated for mCherry‐BCL‐2 (red), GFP‐tBID (green, similar to GFP‐BAX), and cross‐correlation (blue). (B) Cross correlations between BCL‐2 wild‐type /mutants and tBID untreated (‐) and tBID treated with 200 nM VEN (+). For statistical analysis, Welch's t‐tests with Holm–Bonferroni correction were used to compare the different samples to untreated WT that revealed no significant difference (black). Within each genotype, comparisons between untreated and 200 nM VEN‐treated cells are shown and color‐coded by sample. For each sample, between 14 and 25 cells were evaluated on at least 3 different days. (C) Cross‐correlation of mCherry‐BCL‐2 mutants with ‐GFP‐tBID without VEN, minus treated with 200 nM VEN. For statistical analysis, a two‐way ANOVA (factors: genotype and treatment, Holm–Bonferroni corrected) was used to assess the combined effects of genotype and treatment and their interaction.

Here, we used scanning fluorescence cross correlation spectroscopy (SFCCS) to investigate the molecular mechanisms by which BCL‐2 mutations contribute to the interaction of mCherry‐BCL‐2 with GFP‐tBID directly in the mitochondria of living HCT AKO cells and how their binding was affected by VEN (Figure 5A). In the absence of VEN, all BCL‐2 mutants showed cross‐correlation values similar to the values of the wild‐type condition (Figure 5B). Notably, although not significant, G101V showed a trend toward increased interaction with tBID, as shown by the higher cross‐correlation values. Upon VEN treatment, the cross‐correlation between tBID and BCL‐2 wild‐type was decreased, indicating efficient disruption of the complex (Figure 5B). In contrast, the impact of VEN on the reduction of the cross‐correlation levels of all BCL‐2 mutants tested in this assay with tBID was significantly smaller compared to wild‐type (Figure 5B, black p‐values), indicating a reduced ability of VEN to displace tBID from complexes with the BCL‐2 mutants in the mitochondria of cells.

When comparing the change in cross‐correlation under untreated conditions vs. treated with VEN (Figure 5C), R129L and R139H mutants exhibited the lowest decrease in cross‐correlation upon VEN treatment, together with V156D (Figure 5C), suggesting that their binding to VEN is compromised. We performed a two‐way ANOVA, resulting in all mutations except G101V having significant differences compared to wild‐type BCL‐2. The lack of significant change relative to WT for G101V resulted from its higher cross‐correlation with tBID in the absence of VEN. G101V also presented the largest drop in cross‐correlation of all mutants, more similar to the BCL‐2 wild‐type. Although VEN treatment decreased BCL‐2/tBID interactions for this mutant, the cross‐correlation levels remained significantly higher than for the WT, in line with the inability of VEN to disrupt BCL‐2 G101V complexes with tBID as efficiently as the WT. Collectively, these results show that cancer‐related mutations in BCL‐2 have dual effects. First, they alter BCL‐2‐binding affinity for tBID. Second, the BCL‐2 mutations influence the sensitivity of cells to VEN‐induced displacement of tBID from complexes with BCL‐2 at the mitochondria of living cells. Similar effects likely apply to other BH3‐containing interaction partners that bind through the same interface, including BAX and BIM, of relevance in the context of CLL.

### 2.6 Cell Death Assays Define VEN Resistance in Mutants G101V, D103Y, R129L, and V156D

**FIGURE 6:**
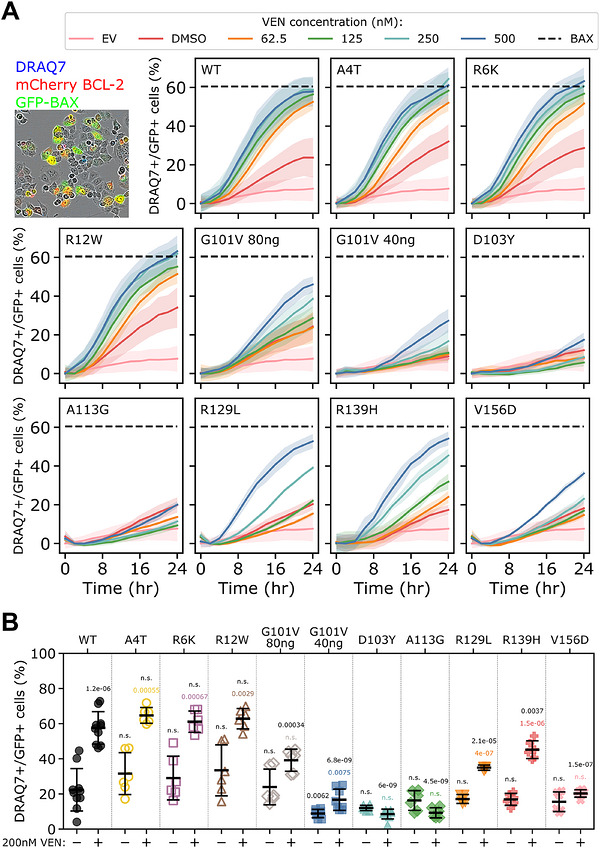
Venetoclax (VEN) cannot block BAX inhibition in cells with cancer‐related BCL‐2 mutations. (A) Left: Example Incucyte image of HCT AKO cells co‐transfected with GFP‐BAX (green), mCherry BCL‐2 (red), and cell death marker DRAQ7 (blue). Rest: Dose‐dependent cell death kinetics of HCT AKO cells co‐transfected with GFP‐BAX and mCherry‐BCL‐2 wild‐type or mutants treated either with DMSO or VEN concentrations ranging from 62.5 to 500 nM. For all samples except “G101V 40 ng”, 80 ng of mCherry‐BCL‐2 DNA was used for transfection. For “G101V 40 ng” 40 ng of mCherry‐BCL‐2 DNA was used for transfection. Cells were seeded 2 days prior to the experiment in a 96‐well plate, co‐transfected, and cell death was observed for 24 h. The marker for cell death used was DRAQ7. BAX‐only positive control is indicated as a black dashed line; empty vector negative control is shown in pink. *n* = 6–12 with 3–6 biological and 2 technical replicates each. (B) Quantification with means and standard deviations of (A) without treatment (‐) and (+) treated with 250 nM VEN. For statistical analysis, Welch's t‐tests with Holm–Bonferroni correction were used to compare samples to untreated WT (black). Within each genotype, comparisons between untreated and 250 nM VEN‐treated cells are shown and color‐coded by sample.

To quantify the ability of the cancer‐related BCL‐2 mutants to resist apoptosis, we conducted a high‐throughput dose‐dependent cell death assay, with HCT AKO cells co‐expressing GFP‐BAX and wild‐type BCL‐2 or cancer‐related mutants treated or not with VEN (Figure 6A). We matched the G101V expression with the other variants as mentioned before (Figure S4B; Figure 6A **“G101V 40 ng”**), for reference, the one with increased expression is also present (Figure 6A **“G101V 80 ng**”). Expression of GFP‐BAX in HCT AKO cells spontaneously induces apoptotic cell death due to lack of inhibition by pro‐survival BCL‐2 proteins, which can be partially inhibited by co‐expression of BCL‐2. Addition of VEN releases BAX from the inhibitory complexes with BCL‐2, leading to apoptosis induction. Interestingly, only BCL‐2 G101V reduced cell death levels in BAX‐expressing cells significantly compared to WT in the absence of VEN (Figure 6B), indicating increased anti‐apoptotic function of this mutant in line with the previous assays.

In our assay, the induction of cell death by VEN treatment compared to the untreated control reports VEN effectiveness in releasing BAX from inhibition by wild‐type BCL‐2 and inducing apoptosis, as well as the effect of cancer‐related BCL‐2 mutations on this process. Remarkably, all mutants except A113G, A4T, R6K, and R12W exhibited significant levels of resistance toward VEN‐induced cell death when compared to BCL‐2 wild‐type (Figure 6B). Among them, both G101V and D103Y were much less susceptible to VEN‐induced apoptosis in this system compared to wild‐type BCL‐2, thus confirming that the G101V and D103Y mutations confer the strongest VEN resistance. Although to a lesser extent, the R129L, R139H, and V156D mutants also presented increased resistance to apoptosis induction via VEN in this assay, despite their similar ability to inhibit BAX‐mediated cell death compared to the wild‐type protein.

### 2.7 Cancer‐Associated BCL‐2 Mutants Retain Their Ability to Inhibit BAX‐Induced MOMP, but tBID and BAX are Less Displaced by VEN

**FIGURE 7:**
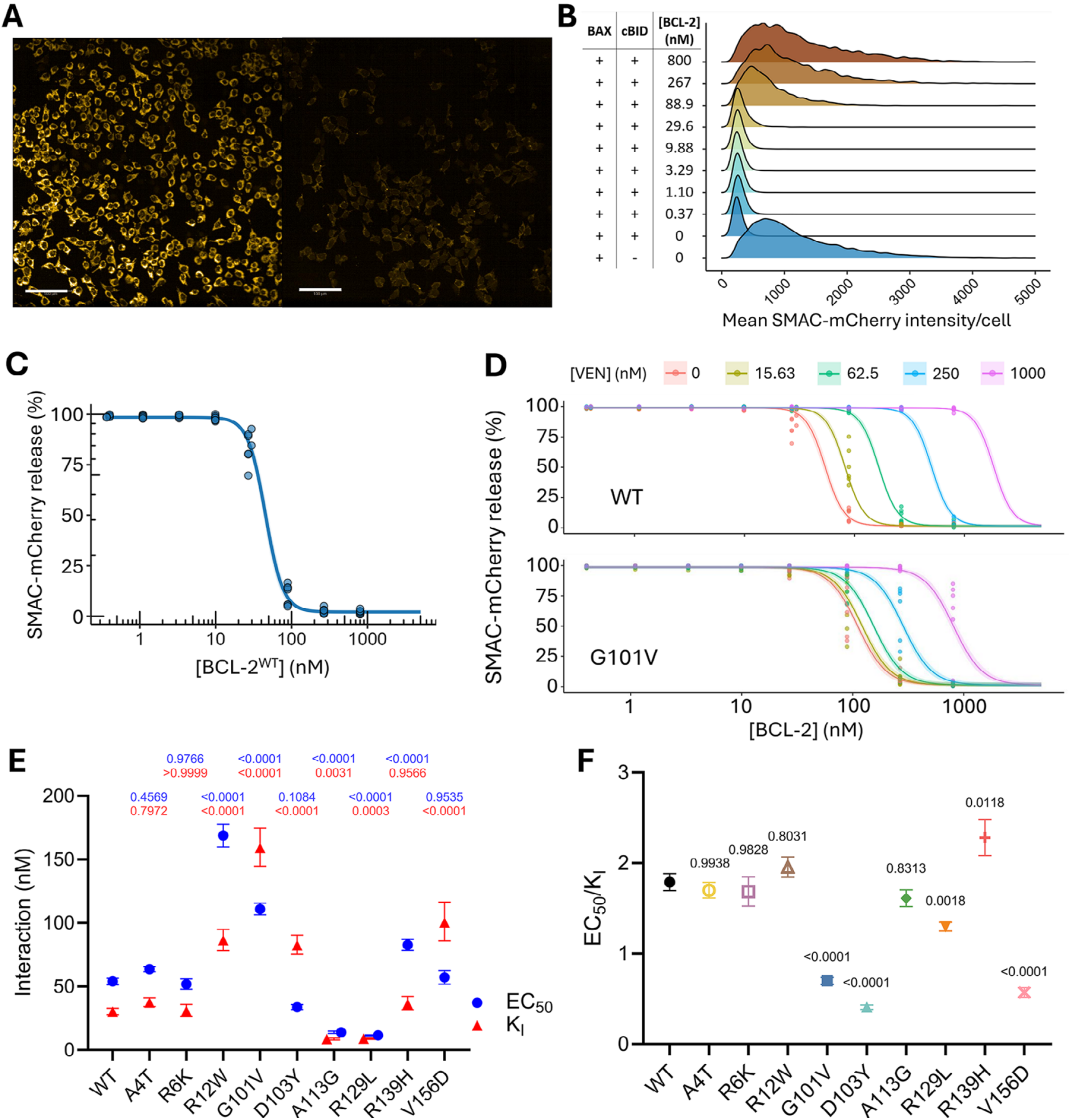
Inhibition of cBID/BAX‐induced MOMP by BCL‐2 is less efficiently disrupted by VEN in cancer‐related BCL‐2 mutations. (A) Representative microscopic acquisitions of SMAC‐mCherry fluorescence in permeabilized baby mouse kidney (BMK) BAX/BAK double‐knockout (DKO) cells without (left) and with the addition of recombinant cBID. The white scale bar indicates 100 µm. (B) Kernel density estimate of mean SMAC‐mCherry intensity per BMK DKO cell of a single representative experiment looking at the cBID‐induced, BAX‐mediated release in the presence of various concentrations of wild‐type (WT) BCL‐2^∆TMD^. (C) A representative fit curve of the percentage of SMAC‐mCherry release showing EC_50_ (half‐maximal effective concentration) for BMK DKO cells with the addition of recombinant BAX, cBID and various concentrations of recombinant wild‐type BCL‐2^∆TMD^. The blue line corresponds to averages of *n* = 9 experiments. The 95 % confidence interval of fit is shown, and blue points indicate individual replicates. (D) Representative fit curve of the percentage of SMAC‐mCherry release showing EC_50_ (half‐maximal effective concentration) for BMK DKO cells with the addition of recombinant BAX, cBID, various concentrations of VEN (represented by different colors of fitting, replicates, and 95 % confidence interval), and various concentrations of recombinant wild‐type BCL‐2^∆TMD^ (upper) or BCL‐2^∆TMD^ mutant G101V (lower). The respective BCL‐2^∆TMD^ mutations (wild‐type and G101V) are indicated in the lower left corner of each graph. The fittings were performed on average SMAC‐mCherry release values obtained from *n* ≥ 3 experiments. The 95 % confidence interval of fit is shown, and colored points indicate individual replicates. (E) Quantification of EC_50_ of the SMAC‐mCherry release in BMK DKO cells with the addition of recombinant BAX, cBID, and various concentrations of recombinant wild‐type and mutant BCL‐2^∆TMD^ proteins, as well as the K_I_ values of the effect of VEN thereon. The p‐values of the statistical analysis of EC_50_ values of different recombinant BCL‐2^∆TMD^ proteins (one‐way ANOVA, Dunnett's multiple comparisons test, blue) as well as the K_I_ values of VEN (two‐way ANOVA, Šidák multiple comparisons test, red) are indicated above the mean values. (F) Quantification of the ratio of the EC_50_ of the SMAC‐mCherry release over the K_I_ of VEN on said process as a composite resistance score. The indicated p‐values were obtained via one‐way ANOVA and Dunnett's multiple comparisons test comparing all conditions to the wild‐type condition.

To directly examine the effect of cancer‐related BCL‐2 mutations on the ability of VEN to induce MOMP, we monitored SMAC‐mCherry release from mitochondria upon addition of recombinantly expressed and purified BCL‐2^∆TMD^, cBID, and BAX to BAX/BAK double‐knockout (DKO) baby mouse kidney (BMK) cells permeabilized with digitonin and treated with or without VEN. This approach allows careful control of concentrations of the proteins analyzed and of VEN to quantitatively analyze MOMP induction. In the absence of cBID, SMAC‐mCherry remained stably localized within mitochondria, confirming that BAX was not spontaneously activated under these conditions (Figure 7A left). Upon addition of cBID, BAX activation led to a marked decrease in mitochondrial SMAC‐mCherry fluorescence, indicative of MOMP (Figure 7A right). As expected, increasing concentrations of wild‐type BCL‐2^∆TMD^ effectively suppressed MOMP, resulting in retention of SMAC‐mCherry in mitochondria (Figure 7B). For BCL‐2^∆TMD^, we determined the half maximal effective concentration (EC_50_) in this assay to be 54 nM for 20 nM BAX and 2 nM cBID (Figure 7C), in line with previous studies [44]. Through nonlinear Bayesian fitting of the data, we calculated the EC_50_ of MOMP inhibition by wild‐type and mutant BCL‐2^∆TMD^ and the respective K_I_ of VEN on them. The fitting of the data for wild‐type BCL‐2^∆TMD^ and BCL‐2^∆TMD^ G101V is shown in Figure 7D, while the fitting of all the other evaluated BCL‐2 mutants is shown in Figure S5. While BCL‐2^∆TMD^ V156D suppressed MOMP similar to BCL‐2^∆TMD^ (Figure 7E), mutants D103Y, A113G, and R129L showed increased inhibitory activity. In contrast, mutants G101V and R139H showed significantly impaired inhibition of cBID/BAX with an EC_50_ of 111 and 83 nM, respectively.

In line with the cell death assays, in the presence of VEN, the inhibition of SMAC‐mCherry release by wild‐type BCL‐2^∆TMD^ was de‐repressed by VEN in a concentration‐dependent manner with a K_I_ of 30 nM (Figure 7D,E). We found that the K_I_ of BCL‐2^∆TMD^ G101V, D103Y, and V156D was significantly increased when compared to wild‐type BCL‐2^∆TMD^, indicating that higher concentrations of VEN were needed to de‐repress the inhibition of BAX‐mediated MOMP by these mutants. BCL‐2^∆TMD^ A113G and R129L showed significantly lower K_I_ values when compared to wild‐type BCL‐2^∆TMD^, while that of R139H was comparable to that of BCL‐2^∆TMD^.

We consider that the effectiveness of VEN on patient outcome is determined by both the potency by which BCL‐2 proteins sequester pro‐apoptotic BCL‐2‐family members, thereby preventing BAX‐mediated MOMP (identified in our assay as an EC_50_), as well as the potency by which VEN antagonizes this function of BCL‐2 protein (identified in our assay as a K_I_). Since both the EC_50_ and the K_I_ are different among wild‐type BCL‐2^∆TMD^ and the cancer‐related mutants, we performed a dual‐marker resistance assessment and calculated the ratio of the EC_50_ for MOMP inhibition by BCL‐2^∆TMD^ over the K_I_ for VEN repressing this process. This yielded the ratio EC_50_/K_I_ as a composite resistance score that provides insights into the relative effectiveness of VEN to antagonize the function of the different BCL‐2 proteins in light of their altered potency to inhibit BAX. In case a BCL‐2 mutant is more resistant to VEN, it is expected that a higher VEN concentration would be required to interfere with BCL‐2's ability to suppress SMAC release, thereby leading to a higher K_I_ and thus a lower EC_50_/K_I_ ratio than BCL‐2^∆TMD^. This analysis revealed that particularly BCL‐2^∆TMD^ G101V, D103Y, and V156D displayed a profound reduction in the EC_50_/K_I_ compared to BCL‐2^∆TMD^ (Figure 7F). Consistent with our results, the EC_50_/K_I_ of R129L was slightly decreased compared to BCL‐2^∆TMD^, while we did not observe a significant decrease in EC_50_/K_I_ for A113G and R139H compared to BCL‐2^∆TMD^. We did not observe any significant effects on the EC_50_/K_I_ of BCL‐2^∆TMD^ A4T, R6K, and R12W when compared to BCL‐2^∆TMD^. Ultimately, this assay reinforces our results that G101V, D103Y, R129L, and V156D confer stronger VEN‐resistance to BCL‐2.

### 2.8 Sonrotoclax (SON) Partially Overcomes VEN Resistance in BCL‐2 Mutants

**FIGURE 8:**
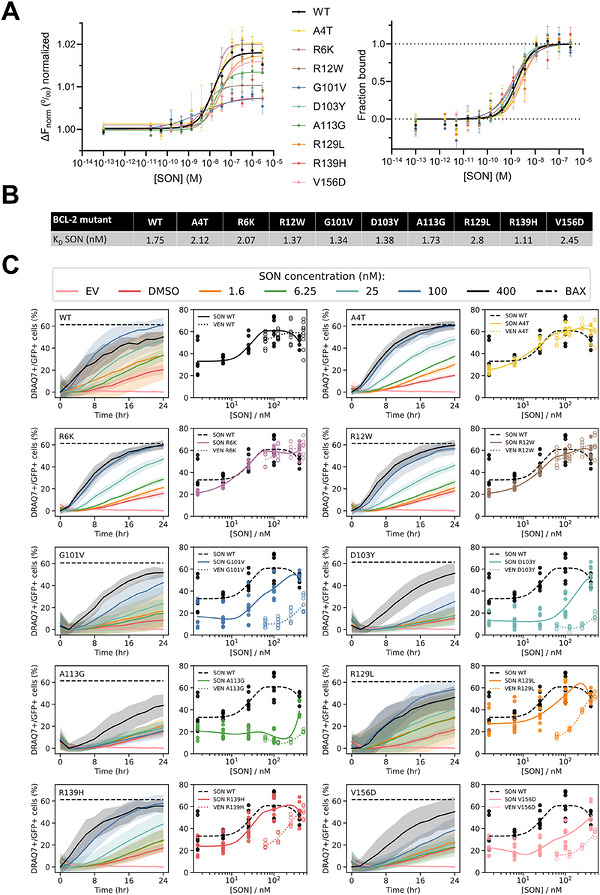
Sonrotoclax (SON) binds with high affinity to all cancer‐associated BCL‐2 mutants and is more effective than VEN in antagonizing VEN‐resistant BCL‐2 mutants. (A) Right: MST binding curves displaying observed thermophoretic shift in function of sonrotoclax (SON) concentration. Fitted curves illustrate the interaction between purified and RED‐labeled purified 6xHis‐tagged BCL‐2^∆TMD^ (10 nM) and SON. The y‐axis (ΔF_norm_) represents the ratio of normalized fluorescence (F_norm_), which is again normalized using the ΔF_norm_ corresponding to the lowest respective BCL‐2^∆TMD^ protein concentration. The x‐axis depicts a logarithmic scale of the corresponding SON concentration. Data points represent mean ± SEM for *n* = 3 biological replicates, each containing 1–3 technical replicates. Left: corresponding data analyzed. The y‑axis (fraction bound) represents the F_norm_ transformed using the unbound and bound plateau phases of the binding curve. (B) Table showing the results of the performed MST experiments evaluating the interaction of BCL‐2^∆TMD^ with venetoclax (VEN) and SON in K_D_. NA: data not available or not applicable for this condition. (C) Columns 1 and 3 show dose‐dependent cell death kinetics of HCT AKO cells co‐transfected with GFP‐BAX and either mCherry‐BCL‐2 wild‐type (WT) or the indicated mutants, followed by treatment with DMSO or increasing SON concentrations ranging from 1.6 to 400 nM. Cells were seeded in 96‐well plates two days before imaging, co‐transfected, and monitored for DRAQ7‐positive cell death over 24 h. BAX‐only positive control is shown as a black dashed line, whereas the empty‐vector negative control is shown in pink. Columns 2 and 4 show the corresponding dose‐response curves derived from the final time point, with individual measurements shown as points and B‐spline fits overlaid. For comparison, data from VEN‐treated samples (Figure 6) are included as hollow points with dotted B‐spline fits. *n* = 6–12 with 3–6 biological and 2 technical replicates are shown each.

Next‐generation BCL‐2 inhibitors, such as sonrotoclax (BGB‐11417; in the following referred to as SON), are currently being developed to antagonize VEN‐resistant BCL‐2 mutants. To explore the effects of the BCL‐2 mutations on SON efficacy, we evaluated the direct binding of SON to the recombinant BCL‐2^∆TMD^, wild‐type or mutant, via MST, similarly to the experiments with VEN. We found that all mutant BCL‐2 proteins bound SON with a K_D_ similar to that of wild‐type BCL‐2 (1.75 nM), ranging from ∼1 to 3 nM (Figure 8A,B), revealing an overall higher binding affinity independent of the cancer‐associated mutations.

We also examined the cell death‐inducing activity of SON (Figure 8C), similarly to the experiments described in Figure 6. Overall, SON was equally effective as VEN in antagonizing wild‐type BCL‐2 and could antagonize BCL‐2 mutants (i.e., A4T, R6K, R12W) not resistant to VEN as potently as wild‐type BCL‐2. Remarkably, SON was considerably more potent than VEN in antagonizing the BCL‐2 mutants displaying resistance to VEN (i.e., G101V, D103Y, A113G, R129L, R139H, V156D), demonstrating its ability to target these VEN‐resistant mutants. Yet, the efficacy of SON against VEN‐resistant BCL‐2 mutants was heterogeneous. While SON was effectively antagonized by R129L and R139H mutants, it was less effective in targeting G101V, D103Y, A113G, and V156D mutants compared to wild‐type BCL‐2. Importantly, A113G appeared highly resistant to SON, with only the highest concentration being capable of inducing BAX‐mediated cell death.

## 3 Conclusions

VEN has emerged as a highly potent and selective BCL‐2 inhibitor used as medication in the clinic as Venclexta and Venclyxto to treat CLL patients [8, 9, 10, 11]. Yet resistance to VEN therapy, particularly in relapsed or refractory patients, remains a significant obstacle. In this study, we systematically investigated a panel of BCL‐2 point mutations identified in patients with a variety of hematological cancers, including CLL, DLBCL, and FL. Some of the mutations, like G101V and D103Y, have previously been associated with resistance to VEN treatment [24, 26, 27, 28]. However, a comprehensive evaluation of BCL‐2 function, VEN binding, and structural dynamics across a broader set of mutants, including A113G, R129L, R139H, and V156D, was lacking.

To evaluate the impact of the cancer‐related mutations on VEN binding to BCL‐2 and to dissociate it from complexes with pro‐apoptotic proteins, we employed a combination of in vitro and in cell experimental approaches. We consistently found that all tested BCL‐2 mutants retained the ability to bind a BIM BH3 peptide in vitro and tBID and BAX in cells, suggesting that the core BH3‐binding groove remains functionally competent (Figures 1, 4, and 5). However, the extent to which VEN could dissociate BCL‐2 from complexes with pro‐apoptotic proteins varied significantly among the mutants. The decreased displacement of pro‐apoptotic proteins observed for mutants G101V, D103Y, R129L, and V156D was consistent across assays. We also observed that G101V, D103Y, R129L, and V156D were less able to bind VEN directly (Figure 2). These observations confirm previous results focused on G101V and D103Y [15, 31, 32] and reveal similar impairments for R129L and V156D.

The HDX‐MS analyses reveal dynamic changes to BCL‐2's protein scaffold as a result of the mutations, which could underline the differences in VEN binding. The BCL‐2 mutants that exhibited impaired VEN binding (G101V, D103Y, A113G, R129L, R139H, V156D) displayed more pronounced and widespread structural changes relative to the wild type, often extending into the canonical BH3‐binding groove and surrounding helices (Figure 3). The affected region is distal to A113G, R129L, R139H, and V156D, indicating allosteric effects. Recently, Sun et al. observed a multitude of structural dynamic changes as a result of VEN binding to BCL‐2 using HDX‐MS [45]. A recent study also reported that VEN‐resistance mutations in BCL‐2 can propagate conformational changes across the BCL‐2 scaffold, affecting VEN binding indirectly [46]. We speculate that the abundant structural shifts in G101V, D103Y, R129L, R139H, and V156D contribute to the decrease in VEN binding (V156D), in the VEN‐induced dissociation from their interaction with pro‐apoptotic proteins (A113G, R129L, R139H), or both (G101V, D103Y). HDX‐MS revealed mutation‐associated changes in structural dynamics extending to additional regions of the VEN‐binding pocket, supporting a model in which V156D reduces VEN binding through a combination of local pocket perturbation and broader dynamic remodeling of BCL‐2. Remarkably, all mutants with decreased VEN binding also showed altered VEN‐induced T_m_, reinforcing the link between structural dynamics and drug resistance. These findings underscore the importance of structural plasticity and allosteric communication within BCL‐2.

To test whether these structural effects translate into functional consequences in cells, we performed FLIP and SFCCS analyses in living cells to directly monitor the dynamics of BCL‐2 interactions at mitochondria using a reductionist system based on tBID and BCL‐2. In the apoptotic interaction landscape of CLL, BIM plays a more prominent role [47]. Also, the displacement of BIM from BCL‐2 by VEN appears less effective than that of tBID due to a double‐bolt lock interaction involving the C‐terminal sequence of BIM [4]. Yet, although distinct from the BCL‐2 interactome in primary cancer cells, our cellular model with tBID is deemed valuable as it generally recapitulates the effect of VEN on BH3‐into‐groove interactions. We find that not only mutations within, but also outside of the BH3 domain of BCL‐2, can lead to changes in binding to tBID at the mitochondria as well as to the dissociation of these complexes by VEN (Figures 4 and 5) [40, 41]. BCL‐2 mutants G101V and D103Y presented a significant increase in their ability to prevent BAX‐mediated cell death under untreated conditions, whereas R129L showed a small decrease relative to wild‐type levels (Figure 6). Addition of VEN revealed that, besides the previously known G101V and D103Y, mutations A113G, R129L, R139H, and V156D confer resistance to VEN‐induced apoptotic cell death. The role of G101V, D103Y, R129L, and V156D as resistance mutations is further reinforced by their composite resistance score of cBID/BAX‐mediated SMAC‐mCherry release and the effects of VEN thereon at different BCL‐2^∆TMD^ concentrations (Figure 7). Of note, potential changes in protein abundance among BCL‐2 mutants, in addition to altered VEN‐binding properties, will impact the eventual resistance outcomes to VEN in primary tumors, which we did not explore in this study.

We find distinct VEN‐resistance phenotypes in BCL‐2 A113G, R129L, R139H, and V156D. BCL‐2 V156D shows decreased binding to VEN and decreased VEN‐induced displacement of pro‐apoptotic BCL‐2 family members without altering their affinity for them (Figure 1E) [46]. Mutation R129L also results in VEN resistance of BCL‐2, characterized by a slightly decreased binding to VEN, increased affinity for BIM, and a slightly decreased VEN‐induced displacement of tBID. In contrast, BCL‐2 mutations A113G and R139H do not affect the direct VEN binding, but they did decrease the VEN‐induced displacement of BAX and/or tBID as well as increase affinity toward BIM and/or BID (Figure 2B,C). Of these mutants, R129L and V156D show the most consistent resistance, while the resistance conferred by A113G and R139H was more dependent on the experimental conditions and could be related to the inclusion or not of the membrane environment in the assay.

Strikingly, CLL patients are often observed with multiple BCL‐2 mutations, with the appearance of one VEN‐resistance mutation showing selective pressure toward the other VEN‐resistance mutations in BCL‐2 [24]. However, we opted for the evaluation of the single BCL‐2 mutations to isolate the various functional effects of every individual mutation, thereby disregarding potential synergism between BCL‐2 VEN‐resistance‐associated mutations. Another methodological limitation in this study is the use of recombinant BCL‑2^ΔTMD^ lacking the TMD required for mitochondrial membrane anchoring, which may not fully recapitulate the behavior of the full‐length protein in the cell. Given that several cancer‑associated mutations elicit long‑range allosteric effects in our HDX‑MS measurements, it is tempting to speculate that the extent of these structural changes may differ in full‑length, membrane‑embedded BCL‑2. Nevertheless, our complementary cell‑based assays using full‑length BCL‑2 support the physiological relevance of the observed mutation‑specific phenotypes.

Interestingly, BCL‐2 A113G, R129L, R139H, and V156D were observed in both DLBCL and FL patients with links toward increased disease progression in the absence of VEN‐treatment [34, 35]. Our observation that these VEN‐resistant mutants retain BH3 binding but exhibit impaired VEN displacement is consistent with the notion that mutations preserve or even increase anti‐apoptotic function while selectively disrupting interaction with VEN. Such preexisting VEN‐resistance mutations could explain why malignancies such as DLBCL and FL, which are highly BCL‐2‐dependent and mutation‐prone due to BCL‐2 translocation t(14;18), do not respond to VEN to the same extent as CLL patients do. However, other resistance mechanisms, such as the upregulation of MCL‐1/BCL‐XL or mutations in BAX, are likely to complement one another in the disease context, especially in the presence of mutations in BCL‐2 that prevent effective inhibition by VEN [14, 24, 36, 48, 49]. Recent clinical sequencing data from CLL patients treated with VEN indicated clonal evolution, particularly of G101V, under VEN pressure, where low‐frequency BCL‐2 mutations can expand over time and co‐occur with broader genomic alterations linked to therapy resistance [28]. Thijssen et al. emphasized that variability both between patients and within individual patients is biologically significant. They suggested that several resistant clones can develop in parallel, each likely enhancing cancer cell survival in the presence of VEN. Because the subclones observed at relapse did not appear to have a general proliferative advantage, the rise of polyclonal resistance mechanisms may be a hallmark of resistance acquired during BH3‐mimetic therapy [20]. In addition, resistance toward VEN might also be a result of transformation toward Richter syndrome as a result of TP53 or BTG1 mutations or deletions of CDKN2A [14].

To target VEN‐resistant BCL‐2 mutants, next‐generation BH3 mimetics such as SON have been developed. Recently, SON was found to have enhanced potency and selectivity, including activity against VEN‐resistant mutants such as G101V [50]. Here, we validated that SON could bind to both wild‐type and mutant BCL‐2^ΔTMD^ proteins with high affinity (K_D_ of 1–3 nM), irrespective of the BCL‐2 mutations associated with VEN resistance. Moreover, SON was overall more effective than VEN in antagonizing VEN‐resistant BCL‐2 mutants. However, different BCL‐2 mutants (G101V, D103Y, V156D, and particularly A113G) remained less susceptible to SON treatment compared with wild‐type BCL‐2, with A113G being highly resistant to SON. These findings indicate that certain resistance‐associated mutations can still attenuate SON efficacy in cells. The discrepancy between biochemical and cellular assays suggests that preserved binding to truncated BCL‐2 is not necessarily sufficient to predict the full apoptotic response in cells, which may be relevant for guiding further drug optimization. Further work will be needed to understand the molecular basis of the divergent efficacy of SON to antagonize VEN‐resistant BCL‐2 mutants in cellular contexts.

Our integrative strategy combining biophysical and cellular approaches delineates how selected BCL‐2 mutations can diminish VEN sensitivity through diverse mechanisms. This includes impaired VEN binding directly, enhanced anti‐apoptotic function, or structural adaptations that reduce the BH3 mimetic efficacy to displace pro‐apoptotic binding partners. Our expanded analysis confirms the previously reported decreased binding to VEN for BCL‐2 G101V and D103Y mutants and identifies an increased binding of pro‐apoptotic family members for G101V but not D103Y [24]. We also dissect the VEN‐resistance properties of new cancer‐related BCL‐2 variants, such as A113G, R129L, R139H, and V156D, and evaluate how they impact SON efficacy, which warrants further investigation in clinical settings. Understanding the allosteric effects of such mutations is critical for anticipating resistance mechanisms and for guiding the rational design of next‐generation BCL‐2 inhibitors with improved resilience to mutational escape.

## 4 Experimental Section/Methods

### 4.1 Cell Culture

HCT‐116 all BCL‐2 proteins knocked out (HCT AKO) cell line was kindly provided by the authors of [38] and confirmed via western blot and cultivated in McCoy's 5A (modified) Medium (Gibco, 16600082). All cell culture growth media were supplemented with heat‐inactivated fetal calf serum (10 %) and antibiotics (penicillin (1 %) and streptomycin (1 %)) unless specified otherwise. BMK cells with BAX and BAK knockout (BMK DKO) were obtained from Eileen White [51]. All cell lines were always passaged at sub‐confluence and cultivated at 37 °C in the presence of CO_2_ (5 %). Regular mycoplasma tests were conducted to exclude contamination.

### 4.2 Plasmids

For the purification of BCL‐2^∆TMD^ proteins, cDNAs sequence coding for C‐terminally truncated BCL‐2 were cloned into a pET45b(+) plasmid to allow purification using standard His‐purification protocols as previously described [52]. The plasmids for the purification of BCL‐2^∆TMD^ proteins were generated through PCR site‐directed mutagenesis as previously described (Integrated DNA Technologies, Leuven, Belgium) [53]. All utilized constructs were verified by sequencing (LGC Genomics, Berlin, Germany). The primers used for site‐ directed mutagenesis were commercially acquired (Integrated DNA Technologies, Leuven, Belgium). For transient transfection of BCL‐2, the full sequence was cloned into pcDNA3.1‐mCherry, and site‐directed mutagenesis was performed similarly to above. BAX and tBID were cloned into pEGFP.

### 4.3 Protein Purification

Purified BCL‐2^∆TMD^ proteins were generated in BL21 *Escherichia coli*. Cultured bacteria, containing protein expression plasmids, were diluted to a density of approximately 0.2 (A600). A heat shock at 40 °C was applied for 2 h, after which isopropyl β‐D‐1‐thiogalactopyranoside was added (100 µM) to induce protein expression. Then, the bacteria were incubated for 2 h 30 min at 20 °C. Bacteria were harvested by centrifugation at 5000 g for 10 min. The bacterial pellet was resuspended in a lysis buffer (NaCl (150 mM), Tris (10 mM), imidazole (30 mM), glycerol (20%), pH 7.4). Samples were sonicated three rounds at 20 kHz (5 × 10 s) with a 15 min incubation at 4 °C between each sonication round. Subsequently, the bacterial lysate was centrifuged at 35 000 rpm for 40 min. Supernatant was collected and incubated with nickel‐nitrilotriacetic acid resin (Ni–NTA Sepharose^TM^ 6 Fast Flow) for 20 h at 4 °C. A lysis buffer containing imidazole (500 mM) was used to eluate all proteins (wild‐type 6xHis_BCL‐2^∆TMD^ and 9 6xHis_BCL‐2^∆TMD^ mutants). Finally, the purified proteins were dialyzed using a Slide‐A‐LyzerDialysis Cassette G2 (ThermoFisher Scientific) with a cutoff of 10 kDa in phosphate‐buffered saline without Ca^2+^/Mg^2+^ (PBS ‐/‐, Gibco) for 1 h three times successively. Protein aggregates were removed by centrifugation (600 s at 16 100 g at 4 °C) and filtration through a Millex pore filter (Millex, Hydrophilic PTFE membrane, 0.22 µm pore size). Protein concentrations were determined using both the NanoDrop (A280) and Bradford reagent (ThermoFisher Scientific). The identity of purified proteins was verified through immunoblotting as previously described [53]. Furthermore, the quality of each purified protein was evaluated by Coomassie blue staining of the gels using Imperial Protein Stain reagent (ThermoFisher Scientific).

### 4.4 Biolayer Interferometry (BLI)

BLI assays were carried out following a similar general protocol as previously described, with modifications specific to the assessment of indirect binding interactions between recombinant 6xHis‐tagged BCL‐2^ΔTMD^ proteins and VEN [44]. Prior to each assay, the Octet streptavidin (SA) Biosensors were equilibrated in ultrapure Milli‐Q water. An initial 30 s baseline was recorded in the binding buffer (Dulbecco's Phosphate‐Buffered Saline DPBS without Ca^2+^ or Mg^2+^, Tween (0.02 %)). Subsequently, the SA biosensors were loaded using commercial, biotinylated BH3^BIM^ peptides (500 nM, biotin‐Ahx‐DMRPEIWIAQELRRIGDEFNAYYARR) in a loading phase of 220 s. Loading parameters were kept constant across all replicates. Loaded sensors were then washed for 60 s, and a second baseline was measured for 30 s in the same buffer, with the final 5 s used for quantitative analysis. Binding affinities of BCL‐2^ΔTMD^ proteins to immobilized BH3^BIM^ were determined using a dilution series ranging from 6.25 to 250 nM during a 600 s association phase. Then, the dissociation phase (600 s) was measured by incubation in the binding buffer, after which the sensors were fully regenerated through an incubation of 10 s in SDS (0.05 %), followed by a wash using a binding buffer for 60 s. The regenerated sensors were then reutilized to determine the affinity of BH3^BIM^ toward the same BCL‐2^ΔTMD^ protein using the same experimental setup but now pre‐mixed with one of four VEN concentrations (20, 40, 60, and 80 nM). This was repeated for all VEN concentrations. A ligand‐loaded sensor incubated in the binding buffer (+ VEN) during the association phase served as our reference. The generated raw data (software ForteBio Data Acquisition 9.0.0.48) was further processed using the Octet Red 96 analysis software (ForteBio Data Analysis 9.0). All data were processed using the following standardized steps: (1) subtraction of the reference. (2) y‐axis alignment with baseline. (3) inter‐step correction alignment to dissociation. (4) Savitzky‐Golay filtering. (5) Partial local fitting using a one‐by‐one interaction model. The obtained steady‐state results were plotted and analyzed using GraphPad Prism 8.4.2. All affinity measurements were performed in at least three independent experiments using independent protein preparations (n ≥ 3).

### 4.5 Microscale Thermophoresis (MST)

Recombinant 6xHis‐tagged BCL‐2^ΔTMD^ proteins were fluorescently labelled using the RED‐tris‐NTA 2nd Generation His‐tag labelling kit (NanoTemper Technologies, Munich, Germany) according to the manufacturer's instructions. MST binding experiments were performed using the Monolith NT. Automated system (NanoTemper Technologies) in order to assess their direct binding affinity toward VEN. The concentration of fluorescently labelled protein was kept constant at 10 nM across all measurements, while VEN and SON were titrated across a series of increasing concentrations to generate binding curves. All protein samples were thawed and kept on ice prior to use and centrifuged at 13 700 g to remove potential aggregates before use. MST measurements were carried out using the pico‐red laser detection channel, with excitation set to 20 % to reduce photobleaching, and thermophoresis power maintained at 40 %. Premium capillaries (Monolith NT.Automated, NanoTemper Technologies) were used for all measurements. Raw thermophoresis data were processed and visualized using GraphPad Prism software. Binding affinities were calculated by fitting the data using a four‐parameter logistic (4PL) regression model. The normalized fluorescence change (ΔF_norm_) was calculated as the average MST signal between 4 and 5 s. ΔF_norm_ values were further normalized to the response observed at the lowest ligand concentration, allowing for improved comparability of binding curves across different protein variants.

### 4.6 Multi‐angle light scattering (MALS)‐Purification

The purity and homogeneity of BCL‐2^∆TMD^ protein were analyzed using SEC‐MALS with a Superdex 200 Increase 10/300 GL column (GE Healthcare) equilibrated with Tris‐HCl (25 mM) and NaCl (25 mM), pH 8 buffer. Separations were performed in 24 min with a flow rate of 0.8 mL min^−1^ at room temperature. D103Y mutant elution was performed with buffer Tris‐HCl (25 mM) and NaCl (25 mM), pH 8, containing glycerol (20 %) and a flow rate of 0.5 mL min^−1^ in 50 min. The elutions were monitored by using a Dawn Heleos II for MALLS measurement (Wyatt Technology) and an Optilab T‐rEX refractometer for refractive index measurements (Wyatt Technology), and molecular mass calculations were performed using ASTRA software (v6.1.7.17).

### 4.7 Peptide Identification

Prior to conducting HDX‐MS experiments, peptides were identified by digesting undeuterated BCL‐2^∆TMD^ protein using the same protocol and identical liquid chromatographic (LC) gradient as detailed below and performing MSE analysis with a Synapt G2 ESI‐Q‐TOF mass spectrometer (Waters), over the m/z range 100–2000 Da. The collision energy was ramped from 15 to 35 V. Sodium iodide was used for calibration, and Leucine Enkephalin was applied for mass accuracy correction. MSE runs were analyzed with ProteinLynx Global Server (PLGS v3.0.1, Waters, UK) and peptides identified using the primary sequence of BCL‐2^∆TMD^ as a search template. Peptides were individually assessed for accurate identification and were only considered if they had a signal‐to‐noise ratio above 10 and a PLGS score above 7.0. Further, peptides were only considered if they appeared in 3 out of 5 replicate runs for each protein. Data analysis was carried out using DynamX 3.0 (Waters, Milford, MA) software to compile and process raw mass spectral data and generate centroid values to calculate relative deuteration values.

### 4.8 Amide Hydrogen‐Deuterium Exchange Mass Spectrometry (HDX‐MS)

HDX‐MS experiments were carried out as previously described [54]. Isotope labeling of the purified BCL‐2^∆TMD^ proteins was carried out using lyophilized buffer (Tris‐HCl (25 mM) and NaCl (25 mM), pH read 8.0) reconstituted in 99.9 % D_2_O (Euriso‐top). Buffer pH read was adjusted to 7.59 using DCl (Alfa Aesar). D‐exchange buffer was pre‐incubated in a 30 °C water bath, and the D‐exchange reaction was initiated by diluting 500 pmol of protein into deuterated buffer at a 1:10 ratio (90 % final D2O concentration, pH read 7.59). Final concentration of BCL‐2^∆TMD^ was maintained at 10 µM in the D‐exchange reaction. Continuous labeling reaction was incubated for various time points (10 s, 1 min, 5 min, 10 min, 30 min, 100 min, and 48 h), at 30 °C. The D‐exchange reaction was quenched by the addition of pre‐chilled quench buffer (Tris‐HCl (25 mM) and NaCl (25 mM)) with pH adjustment by formic acid to ensure that, after mixing with the sample at a 1:1 ratio, the pH of the final solution reaches 2.5. Then, the reaction was centrifuged at 20 000 g for 1 min on a benchtop‐cooled centrifuge (Sigma), and the supernatant containing BCL‐2^∆TMD^ protein was collected and immediately injected into the LC‐MS system. The quenched sample was then loaded onto a 50 µl sample loop and subsequently digested on a home‐packed immobilized pepsin cartridge (2 mm × 2 cm, Idex, Sigma) maintaiSkned at 16 °C. The resulting peptides were loaded and trapped onto an Acquity BEH C18 1.7 µm, VanGuard C18 Pre‐column, (130 Å, 1.7 mm, 2.1 × 5 mm, Waters) at 100 mL min^−1^ for 3 min using formic acid (0.23 % v v^−1^). Peptides were subsequently separated on a C18 analytical column (UPLC, BEH C18,130 Å, 1.7 mm, 1 × 100 mm, Waters) at 40 mL min^−1^. UPLC separation (solvent A: formic acid (0.23 % v v^−1^), solvent B: formic acid (0.23 % v v^−1^) in acetonitrile) was carried out using a 12 min gradient elution (solvent B (5–50 %)). At the end, solvent B was raised to 90 % for 1 min for column cleaning. Peptide trapping, desalting, and separation were performed at 2 °C. The eluted peptides were ionized by electrospray into the SYNAPT G2 ESI‐Q‐TOF mass spectrometer over the m/z range 100–2000 Da (Waters, UK). All deuterium time points and controls were performed in triplicate (n ≥ 3). The MS parameters were as follows: capillary voltage 3.0 kV, sampling cone voltage 20 V, extraction cone voltage 3.6 V, source temperature 80 °C, desolvation gas flow 500 L h^−1^ at 150 °C. Full deuteration controls were obtained by incubating BCL‐2^∆TMD^ proteins in the deuterated buffer for 48 h at 30 °C. D‐uptake (%) was calculated using the full deuteration control D‐uptake values. Deuterium/Protium back exchange values for our instrumental setup were calculated to be between 20 % and 45 %, depending on peptide composition. These values are consistent with previously reported studies using similar instrumental setups [55]. The data has not been corrected for back exchange and is represented either as absolute D values or as a percent of the full deuteration control [56].

Sequence identification was performed from MS^E^ data of digested undeuterated samples of BCL‐2^∆TMD^ protein using the ProteinLynx Global Server (PLGS v3.0.1, Waters, UK). Peptides were individually assessed for accurate identification and were only considered if they had a signal‐to‐noise ratio above 10 and a PLGS score above 7.0. Furthermore, peptides were only considered if they appeared in 3 out of 5 replicate runs for each protein. The output peptides were then filtered using DynamX 3.0 (Waters, Milford, MA) software, and analysis was carried out to compile and process raw mass spectral data and generate centroid values to calculate relative deuteration values. Gibbs free energy values (ΔG_ex_, kJ mol^−1^) for all residues were determined using the PyHDX software (v0.4.0‐rc1) (Smit et al., 2021 [54]). D‐uptake data from triplicate experiments, for all time points, were input along with 100% deuteration control. Input parameters were set to the following parameters: temperature (303.15 K); pH (7.59); stop loss (5·10^−7^); stop patience (50); learning rate (1·10^5^); momentum (0.5); epochs (2·10^5^); regularizer 1 (1); regularizer 2 (2).

In summary, PyHDX rapidly processes single and multiple HDX‐MS data sets and visualizes residue‐level Gibbs free energies (ΔG) of proteins on linear and 3D structures. ΔG and ΔΔG values were mapped onto linear maps of wild‐type BCL‐2^∆TMD^ and different mutants and on their 3D structure. The values from the two states (ΔΔG) were subtracted and coloured differently (10 kJ mol^−1^, dark purple, increased rigidity; 0 kJ mol^−1^, white, no change; ‐10 kJ mol^−1^, dark green, increased dynamics).

Residue colours are additionally shown as a linear bar, and regions without peptide coverage are coloured in gray. Error bars are covariances. Differential dynamics between wild‐type BCL‐2^∆TMD^ and mutant each shown as differences in ΔG of BCL‐2 wild‐type and mutant (ΔΔG). Regions in purple are rigidifying, and regions in green are dynamic (flexible) in the BCL‐2^∆TMD^ mutant compared to the wild‐type BCL‐2^∆TMD^, respectively, and the 3D structure of BCL‐2^∆TMD^ mutants is colored according to the per‐residue ΔΔG.

### 4.9 Circular Dichroism (CD) Spectropolarimetry

CD spectra were recorded in the far UV range (190–260 nm) using a J‐1500 spectropolarimeter (Jasco) equipped with a six‐position cuvette holder and a Peltier device to regulate temperature (typically 2–18 µM protein to satisfy −5 to −20 mdeg signal range; 1 mm quartz cuvettes). For thermal unfolding analysis, the protein was diluted in buffer (Tris‐HCl (25 mM) and NaCl (25 mM), pH read (8.0)), with final concentrations of 10 and 20 µM for protein and VEN, respectively, containing DMSO (0.2 %). Protein 10 µM containing DMSO (0.2 %) was used as a control sample and was analyzed at the same time as the protein solution in the presence of VEN. Protein spectra were recorded at 221 nm (minima) from 5 °C to 85 °C with data taken every 0.5 °C (CD scale 200 mdeg/1.0 dOD; D.I.T. 0.5 s). Data were analyzed using the Spectra Analysis v.2 software (Jasco); melting temperatures (T_m_) were derived by acquiring the first derivatives of the melting curves, using the calculus function of GraphPad Prism 5.03.

### 4.10 Western Blotting

Two days prior to the experiment, HCT AKO cells were harvested at 70% confluency and subsequently seeded into a 6‐well plate (Greiner, 140675) with a density of 200 000 cells per well. After two days, growth medium was renewed and 50 µL Opti‐MEM (Gibco, 31985062) containing plasmid DNA (500 ng) and Opti‐MEM (50 µL) containing PEI STAR transfection reagent (2.5 µL of 1 µg µL^−1^, CAS 49553‐93‐7) were mixed, incubated for 30 min at room temperature, and added to each well to transiently transfect the cells (final volume 2 mL). 1 day later, cells were lysed in RIPA buffer (NaCl (150 mM), sodium deoxycholate (0.5%), NP‐40 (1%), SDS (0.1%), Tris (50 mM), pH (8.0)) with protease inhibitor. Protein concentration was determined by Bradford protein assay (Bio‐Rad) according to the manufacturer's protocol. Equal amounts of protein (30 µg) were loaded onto a Tris gel (12%, poured) and transferred onto a nitrocellulose membrane using the Turboblot (Bio‐Rad). Blots were blocked with milk (3%) in TBST for 1 h and incubated overnight at 4 °C with the primary antibody mCherry (E5D8F) Rabbit mAb (Cell Signaling, 43590), probed with fluorescent secondary antibody IRDye 800CW Goat anti‐Rabbit (LI‐COR) and developed using a Bio‐Rad ChemiDoc imaging system. Loading normalization was done using α‐Tubulin as a loading control. The intensity was then calculated using the following formula:

$$I_{relative}=\;\log\left(\frac{I}{I_{WT}}\right)$$

### 4.11 Fluorescence Intensity Loss After Photobleaching (FLIP)

To study the intracellular redistribution dynamics of tBID in the presence of different BCL‐2 mutants, we performed FLIP experiments using HCT AKO cells. Two days prior to the experiment, HCT AKO cells were harvested at 70 % confluency and subsequently seeded into an ibidi µ‐Slide 8‐well chamber at a density of 30 000 cells per well and allowed to adhere for 48 h. Transient transfection was then performed using OptiMEM (50 µL) containing mCherry–BCL‐2 (130 ng) and GFP–tBID (20 ng) per well to a final volume of 200 µL, similar to the section above. FLIP was performed both in the absence and presence of VEN (200 nM), using the same protocol.

Imaging was conducted 16–24 h post‐transfection using a Zeiss LSM980 Airyscan confocal microscope equipped with a 60× NA 1.4 oil immersion objective. Cells were first visualized under low intensity to identify suitable candidates with robust mitochondrial signals. Cells exhibiting strong mitochondrial signal and healthy morphology were preferentially selected for analysis. A single cell was selected for each experiment, and a region comprising approximately 80% of the cell area was defined for photobleaching. The cell was conceptually divided into two regions: an 80% bleached region and a 20% unbleached region. The bleached region was photobleached using a laser (488 nm) at 100% intensity for 8 s, followed by a second identical pulse after a 30 s interval.

Immediately after bleaching, both the 80 % and 20 % regions of the cell were imaged together using 0.5 % laser intensity to minimize further photobleaching. The cell was monitored for 300 s at a frame rate of approximately 1 frame per second to capture the redistribution of the fluorescent signal. A progressive decrease in fluorescence intensity in the unbleached region was monitored, consistent with diffusion of GFP–tBID molecules into the bleached area. The normalized fluorescence intensity *I_N_* was normalized to timepoint zero:

$$I_{N}\left(t\right)=\frac{I\left(t\right)}{I\left(0\right)}$$

As controls, two parallel experiments were always included: (1) A bleaching control, where a neighboring unbleached cell was imaged continuously for 300 s under the same 0.5 % laser intensity to account for baseline photobleaching under low‐power imaging conditions. (2) A tBID control, where cells were transfected with GFP–tBID and mCherry only, without BCL‐2 constructs, to assess baseline diffusion behavior of tBID in the absence of BCL‐2.

An unpaired Welch‐corrected two‐tailed t‐test was performed in Python with the following code:

from scipy.stats import ttest_ind
ttest_ind(values_minus[0], values_minus[j], equal_var = False)

And p‐value significance threshold according to Holm–Bonferroni correction was calculated in the following way:

$$p-value\;threshold=\frac{0.05}{N},$$

where N is the number of t‐tests performed in the specific group.

Two‐way ANOVA was performed with the 2 factors that play into the ΔCC in Python with the following code:

import statsmodels.api as sm
from statsmodels.formula.api import ols
df[‘Genotype’] = df[‘Genotype’].astype(‘category’)
df[‘Treatment’] = df[‘Treatment’].astype(‘category’)
model_2way = ols(‘value ∼ C(Genotype) * C(Treatment)’, data = df).fit()
anova2_table = sm.stats.anova_lm(model_2way, typ = 2)

### 4.12 Scanning Fluorescence Cross Correlation Spectroscopy (SFCCS)

Three days prior to the experiment, HCT AKO cells were harvested at 70 % confluency and subsequently seeded into an ibidi µ‐Slide 8‐well chamber with a density of 35 000 cells per well. After two days, transient transfection similar to the section above was performed with DNA (150 ng, mCherry‐BCL‐2 80:20 GFP‐BAX/GFP‐tBID) to a final volume of 200 µL and incubated for 24 h. The SFCCS was performed on a Zeiss LSM980 Airyscan (Carl Zeiss, Jena, Germany) in confocal mode. A 40× NA 1.2 UV–vIS–IR C‐Apochromat water immersion objective was used and adjusted to maximum photon yield before every experiment. Also, before every experiment, the pinhole was adjusted to maximum photon yield. The movement of the detection volume was controlled using the Zeiss LSM software ZEN3. Line mode was used for scanning FCCS measurements. Lines were scanned with 128 × 1 pixels. The correlation and fitting of the correlation curves were performed with a home‐developed software available on GitHub (https://github.com/jaufdermauer/MitoProfiler). For the fit of the measurements, different models have been proposed in the past to describe the diffusion of molecules on the mitochondrial membrane within the focal volume. We tested both 2D diffusion with Gaussian beam profiles and simplified 1D diffusion and found that 1D diffusion yielded a more robust fitting probably due to lower number of free parameters. Previous simulations have reached similar conclusions [41]. The main limitation of this approach is that it assumes a perfectly symmetrical 3D Gaussian laser focal volume, whereas in reality the focal spot is slightly elongated along the z‐axis. However, since mitochondria typically have a smaller diameter than the focal volume (*d_mito_* < *d*_*laser*,*z*_), this deviation becomes negligible as long as the mitochondria remain in focus.

Models:

Convolution:

$$G_{CC}\left(\tau \right)=\int f\left(t\right)\cdot g\left(t+\tau \right)d\tau$$

1D Free Diffusion:

$$G_{1D}\left(\tau \right)=\left(\frac{1}{N}\right)\cdot {\left(1+\frac{\tau }{\tau_{D}}\right)}^{-\frac{1}{2}}$$

2D Elliptical Gaussian:

$$G_{2DGauss}\left(\tau \right)=\left(\frac{1}{N}\right)\cdot {\left(1+\frac{\tau }{\tau_{D}}\right)}^{-\frac{1}{2}}{\left(1+\frac{\tau }{S^{2}\tau_{D}}\right)}^{-\frac{1}{2}}$$

Where $N=C\pi Sw_{0}^{2}$ and $\tau_{D}=4w_{0}^{2}D$

Equation (1) shows diffusion models for SFCCS.

For the cross‐correlation calculation, we used,

(1) $$CC_{gr,g}=\frac{C_{gr}}{C_{gr}+C_{g}}=\frac{G_{gr}\left(0\right)}{G_{g}\left(0\right)}$$

where *CC*_gr,g_ is the cross‐correlation between the green and red channels evaluated vs. the green channel, *C*_i_ are the concentrations, and *G*_i_(0) the correlation functions evaluated at τ  =  0 as explained in Equation (1). All measurements were performed at room temperature with CO_2_ (5 %). Data were included when the curves clearly indicated pure membrane diffusion without cytosolic fraction or mitochondrial movement. Statistical analysis was performed similarly to the section above.

### 4.13 Incucyte Experiments

Two days prior to the experiment, HCT AKO cells were harvested at 70 % confluency and subsequently seeded into a 96‐well plate (Greiner, 2936) with a density of 3500 cells per well. The border wells of the plate were not used but instead filled with PBS to prevent evaporation. After two days, growth medium was renewed and Opti‐MEM (25 µL) (Gibco, 31985062) containing plasmid DNA (100 ng) and Opti‐MEM (25 µL) containing PEI STAR transfection reagent (0.5 µL of 1 µg µL^−1^, CAS 49553‐93‐7) were mixed, incubated for 30 min at room temperature, and added to each well to transiently transfect the cells similar to the section above (final volume 150 µL). After 5 h of incubation at 37 °C and CO_2_ (5 %), the transfection medium was removed, the cells were washed with fresh medium, and new medium containing secondary necrosis cell death marker DRAQ7 Dye (biostatus, D15106) diluted 1:3000 and VEN (AbbVie Inc., LOT 1940704‐0) treatment (ranging from 0 to 500 nM) or DMSO (Sigma‐Aldrich, LOT RNBL9639) was applied. The plates were quickly transferred into the Incucyte, and 4 images per well were taken every 2 h for 24 h. For the analysis of the dose‐dependent cell death assay, we used the built‐in cell‐by‐cell analysis and subsequent cell‐by‐cell classification.

To determine the BCL‐2 G101V DNA amount required to result in equal expression as wild‐type BCL‐2, we titrated G101V with GFP‐empty vector between 100:0 and 40:60 mCherry‐BCL‐2:GFP‐empty. Notably, protein levels were even higher than those of the mCherry‐empty vector, despite BCL‐2's two‐fold smaller size. After 24 h, G101V (40 ng) yielded fluorescence levels comparable to wild‐type BCL‐2. For the evaluation of the brightness, we used cell‐by‐cell analysis followed by exporting the cell‐by‐cell Avg Mean Intensity (Orange) Graph Metrics.

### 4.14 SMAC‐mCherry Release Assay

Baby mouse kidney (BMK) BAX/BAK double‐knockout (DKO) cells, stably expressing SMAC‐mCherry, were seeded at a density of 6000 cells per well in 384‐well PerkinElmer PhenoPlates. All purified recombinant proteins were thawed on ice and centrifuged at 15 000 g to remove potential aggregates 24 h post‐seeding. An eight‐point titration series of purified BCL‐2^ΔTMD^ was prepared in a 384‐well source plate using trehalose‐HEPES buffer (THB) composed of trehalose (300 mM), HEPES‐KOH (10 mM), pH (7.7), KCl (80 mM), EGTA (1 mM), EDTA (1 mM), BSA (0.1 %), and succinate (5 mM). The buffer also contained DRAQ5 (5 µM) nuclear stain, digitonin (0.0025 % w v^−1^d), and either purified BAX alone (20 nM) (negative control) or BAX (20 nM) with cBID (2 nM) (positive control and BCL‐2^ΔTMD^ titration conditions). Where applicable, a four‐point titration series of VEN or an equivalent volume of PBS (vehicle control) was included. Medium was aspirated from the assay plate using a VANTAGE automated liquid handler, followed by two washes with THB (30 µL). Subsequently, protein mixtures from the source plate (25 µL) were transferred to the cells. Cells were incubated for 60–80 min at 37 °C in a humidified incubator with CO_2_ (5 %), after which fluorescence imaging was performed using the Opera Phenix high‐content screening system. Nuclei and mitochondria were imaged using an Opera Phenix microscope, and image analysis was conducted using PerkinElmer Harmony software. Nuclei were detected and segmented using DRAQ5 fluorescence signal, and cytoplasm was segmented based on SMAC‐mCherry fluorescence. For each cell, morphological, intensity, and texture features were extracted from both the DRAQ5 channel in the nuclear segment and the SMAC‐mCherry channel from the cell segment. A random forest (RF) algorithm, using the extracted feature set, was trained against the negative control, “unreleased” wells containing BAX alone, and the positive control, “released” wells containing BAX and cBID. The trained RF model was then used to predict whether a cell was more like a negative “unreleased” control or a positive “released” control. The fraction of cells with released SMAC‐mCherry was calculated by dividing the number of cells predicted to be “released” by the total number of cells in the well for each condition.

### 4.15 Data Analysis

Immunoblot quantification was carried out using FIJI software [38]. MST data were analyzed with MO. Affinity Analysis v2.3 (Monolith). GraphPad Prism (version 11) was used for data visualization, fitting nonlinear regression curves (Sigmoidal, 4PL, concentration as X‐axis), and determining dissociation constants (K_D_) for each experimental condition. BLI measurements were acquired using ForteBio Data Acquisition 9.0.0.48 and analyzed with ForteBio Data Analysis 9.0 (Octet Red 96). The analysis followed a standardized workflow: (1) Subtraction of the naked loaded sensor measurement. (2) Y‐axis alignment (baseline). (3) Inter‐step correction alignment (dissociation). (4) Savitzky‐Golay filtering. (5) Partial local fitting using a one‐by‐one interaction model. Steady‐state data were then plotted and analyzed in GraphPad Prism version 10 using the same nonlinear regression model. Response data were fitted to a steady‐state competition model in line with previous similar evaluations (Equation 2) [27]. The data from our SMAC‐mCherry release assay were fitted via nonlinear Bayesian modelling using Equation (3) as done before [57].

(2) $$R=R_{\max}\cdot \frac{\left[\mathrm{BCL}2\right]-\frac{\left(\left[\mathrm{BCL}2\right]+\left[\mathrm{VEN}\right]+K_{I}\right)\pm \sqrt{{\left(\left[\mathrm{BCL}2\right]+\left[\mathrm{VEN}\right]+K_{I}\right)}^{2}-4\left[\mathrm{BCL}2\right]\left[\mathrm{VEN}\right]}}{2}}{K_{D}+\left[\mathrm{BCL}2\right]-\frac{\left(\left[\mathrm{BCL}2\right]+\left[\mathrm{VEN}\right]+K_{I}\right)\pm \sqrt{{\left(\left[\mathrm{BCL}2\right]+\left[\mathrm{VEN}\right]+K_{I}\right)}^{2}-4\left[\mathrm{BCL}2\right]\left[\mathrm{VEN}\right]}}{2}}$$

Equation (2) shows fitting models for competitive interaction through BLI. *R*  =  response, *R*_max_  = maximal response, *[BCL‐2]*  =   BCL‐2^ΔTMD^ concentration, *[VEN]*  =   VEN concentration, *K*_D_  =   apparent steady‐state binding constant toward BH3^BIM^ peptide, *K*_I_  =  fitted equilibrium binding constant for VEN. *K*_D_ and *R*_max_ for the steady‐state competition model were calculated using a one‐site‐specific binding model on GraphPad Prism 8.4.2 using the data from the 0 nM VEN condition of said experiment. For EC_50_ comparisons in the SMAC‐mCherry release assay, a Wilcoxon rank‐sum exact test was applied due to non‐normal data distribution. Nonlinear Bayesian model fitting for these data was done using the BRMS R package, and Welch's t‐test was performed in base R.


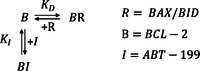

(3) $$F_{\mathrm{rls}}=\;\mathrm{Bottom}+\left(\frac{\mathrm{Top}-\mathrm{Bottom}}{1+{\left(\frac{\left[B_{\mathrm{tot}}\right]}{EC_{50}\left(\frac{\left[I_{\mathrm{tot}}\right]}{K_{I}}+1\right)}\right)}^{n}}\right)$$

Equation (3) shows Nonlinear Bayesian modeling. R = BAX/BID, B = BCL‐2, I = VEN (ABT‐199), *K*_I_  =  fitted equilibrium binding constant for VEN, F_rls_ = fractional occupancy of R measured as the SMAC‐mCherry release. The following set of assumptions was made: [B_free_] ≈ [B_tot_], when [BR] << [B_free_], [B_tot_] = [B_free_] + [BR], [I_free_] ≈ [I_tot_], when [BI] << [I_free_], [I_tot_] = [I_free_] + [BI] [57] (Table 2).

### 4.16 Reagents and Instruments (Tools) Table

**TABLE 2:** Reagents and instruments (tools) table.

Reagent/Resource	Reference or source	Identifier or catalog number
**Software**		
PyHDX	v0.4.2 (Smit et al., 2021 [54])	http://pyhdx.jhsmit.org/
MassLynx	v4.1 (Waters)	Waters Corporation, RRID: SCR_014271
ProteinLynx Global Server (PLGS)	v3.0.1 (Waters)	Waters Corporation
DynamX	v3.0 (Waters)	Waters Corporation
PyMOL	v2.4.0 (Schrödinger)	https://pymol.org/2/; RRID: SCR_000305
GraphPad Prism	v5.03	www.graphpad.com prism/; RRID: SCR_002798
Spectra Manager	v.2.12.0	Jasco (Deutschland GmbH, Germany)
LabSolutions Main	v5.96	Shimadzu Corporation
ASTRA	v6.1.7.17	WAYATT Technology Corporation
**Equipment**		
Wizard SV Gel and PCR Clean‐Up System	Promega	A9281
nanoACQUITY UPLC System with HDX Technology	Waters	Waters Corporation
Synapt G2 Mass Spectrometry instrument	Waters	Waters Corporation
MassPREP Micro Desalting column	Waters	186004032
Pepsin column	Sigma (pepsin) + Idex (cartridge)	P0609 + # 5051IP‐M07021‐005‐05TI
VanGuard C18 Pre‐column	Waters	186003975
C18 analytical column	Waters	186002350
Superdex^TM^ 200 10/300 GL	GE Healthcare	28990944
Dawn Heleos II SEC‐MALS system	Wyatt Technology (Model: WH2‐06)	93117 S.N: 1151‐H2HC
Optilab T‐rEX Refractometer	Wyatt Technology (Model: WTREX‐04)	93117 S.N: 796‐TREX
Nanodrop 2000	Thermo	ND‐2000
Jasco J‐1500	Jasco Inc.	J‐1000 series
Centrifuge 1–16 k	Sigma	D‐37520
Water Bath Digiterm 100	JP SELECTA	3000542
pH meter FiveEasy Plus FEP20	METTLER TOLEDO	B513755638
**Chemicals, enzymes, and other reagents**		
Tris base	Sigma‐Aldrich	T1378
NaCl	Sigma‐Aldrich	7647‐14‐5
Glycerol	Chem‐Lab	CL00‐0706
Phenylmethylsulfonylfluoride (PMSF)	Roth	6367
Dithiothreitol (DTT)	Panreac AppliChem	A1101
Acetonitrile	Merck Millipore	100030
Formic Acid (MS grade)	Sigma–Aldrich	F0507
Leucine Enkephalin (LeuEnk)	Waters	186006013
Sodium iodide	Sigma–Aldrich	383112
Deuterium Oxide (99.9 %)	Euroisotop	D216
DCl 20 %	Alfa Aesar	042407
DMSO		

### 4.17 Use of Artificial Intelligence

All content was generated by the co‐authors. ChatGPT‐5, Claude and Microsoft Copilot have been used as Large Language Models (LLMs) to improve texts.

## Author Contributions

Conceptualization: J.A., I.d.R., J.K., D.A., S.K., A.J.G.S., and G.B. Investigation: J.A., I.d.R., M.E., M.K., J.K., D.A., S.K., A.J.G.S., and G.B. Writing – original draft: J.A. and I.d.R. Writing – review & editing: J.A., I.d.R., M.E., M.K., J.K., L.P.F., D.A., S.K., A.J.G.S., and G.B. Visualization: J.A., I.d.R., and M.E. Formal analysis: J.A., I.d.R., M.E., M.K., and J.K. Data curation: J.A., I.d.R., M.E., M.K., J.K., D.A., S.K., A.J.G.S., and G.B. Methodology: J.A., I.d.R., M.E., J.K., D.A., S.K., A.J.G.S., and G.B. Validation: J.A., I.d.R., M.E., J.K., D.A., S.K., A.J.G.S., and G.B. Funding acquisition: J.A., I.d.R., L.P.F., D.A., S.K., A.J.G.S., and G.B. Resources: L.P.F., D.A., A.J.G.S., and G.B. Project administration: J.A., I.d.R., A.J.G.S., and G.B. Supervision: D.A., S.K., A.J.G.S., and G.B.

## Funding

This work was funded by the Deutsche Forschungsgemeinschaft (DFG, German Research Foundation) – 455784452, 414786233, 269925409, and 471011418 (J.A., A.J.G.S., M.K., L.P.F.); the European Research Council (ERC) under the European Union's Horizon 2020 research and innovation program (grant agreement No 817758) (J.A., A.J.G.S., M.K.); Sonderforschungsbereich‐Geschäftszeichen, 455784452 A06 and Z02 (L.P.F.); A project grant (A05) by the Center for Molecular Medicine Cologne (L.P.F.); the Research Foundation—Flanders (FWO) (G081821N and G094522N) (G.B. and S.K.), the KU Leuven Research Council (C14/19/099, AKUL/19/34 and C14/25/131)(G.B.); and the Central European Leuven Strategic Alliance (CELSA/23/031 and CELSA/23/032) (G.B.). FWO Scientific Research Network CaSign (W0.014.22N) (G.B.); A FWO PhD fellowships (1131322N|1131324N) (I.d.R.), and the Canadian Institutes of Health Research Project Grant (FRN185962) (D.A.).

## Conflicts of Interest

The authors declare no conflicts of interest.

## Supporting information




**Supporting file**: advs76693‐sup‐0001‐SuppMat.docx

## Data Availability

All data of this manuscript are publicly available via KU Leuven Research Data Repository: https://doi.org/10.48804/ZMV1QJ.
