# Supplementary material for: Cancer‐Associated BCL‐2 Mutants Reveal Mechanisms Towards Venetoclax Resistance
Source: Adv Sci (Weinh). 2026 Jul 24;13(57):e76693. doi: 10.1002/advs.76693 (PMC13398141; doi:10.1002/advs.76693)
Supplement: Supplementary file 1 [file ADVS-13-e76693-s001.docx]

Supplementary Material

**Cancer-associated BCL-2 mutants reveal mechanisms towards venetoclax resistance**
*Jonas Aufdermauer^,#^, Ian de Ridder^,#^, Mahjoobeh Ehsani, Marasim Khan, Justin Kale, Lukas Frenzel, David Andrews, Spyridoula Karamanou, Ana J. Garcia-Saez* and Geert Bultynck**

**Figure S1: Biolayer interferometry (BLI) binding curves of the BCL-2 mutants not presented before.**

**A-H**: Additional data of the performed competitive, indirect interaction analysis through BLI of the BCL-2 mutants that were not depicted in Figure 1. Biosensors were loaded with BH3^BIM^ peptides and binding affinities of purified BCL-2^∆TMD^ proteins to immobilized BH3^BIM^ were determined in the presence of various VEN concentrations. The respective BCL-2^∆TMD^ mutations are indicated in the lower left corner of each graph. Binding curves illustrate the interactions between BH3^BIM^ and recombinant mutant BCL-2^∆TMD^ proteins incubated with various VEN concentrations (different colors), which are displayed as average steady state responses determined at the end of each association phase (595-600 s). The steady state values were fitted using Equation 1 to obtain K_D_ for the interaction between BH3^BIM^ and BCL-2^∆TMD^ and K_I_ values for VEN. Each data point reflects the mean ± S.D. for N ≥ 3.

**Figure S2: Direct binding of venetoclax (VEN) to wild-type BCL-2^∆TMD^.** Measured fluorescence of RED-labeled recombinant 6xHis-tagged BCL-2^∆TMD^ (10 nM) in the presence of a gradient of VEN concentrations (0 - 1 µM) are represented as MST traces experiment. Within the graph, the blue shaded (cold) area signifies the data point used for normalization of mean fluorescence, while the red shaded (hot) area indicates the data points used for the quantification of the thermophoretic shift in function of VEN concentration. The dotted vertical lines indicate the time points when the infrared laser was activated (right) and deactivated (left).

**Figure S3:**

**A:** Linear representation of the Gibbs free energies (ΔG, kJ/mol) of recombinant BCL-2 wild-type and mutants, colored according to the per residue value. The respective mutations in each of the mutants are shown right of each linear representation. Annotation of the functional domains (blue) and the secondary structures (grey) within the BCL-2^∆TMD^ protein is shown above. Low ΔG values (red) correspond with high deuterium update and therefore high flexibility or solvent accessibility, while high ΔG values (purple) correspond with low deuterium uptake and therefore low flexibility or solvent accessibility.

**B:** The deuterium-uptake of the indicated peptides obtained from both wild-type BCL-2^∆TMD^ and BCL-2^∆TMD^ D103Y was determined, expressed as a percentage of the deuterium-uptake relative to the maximum possible deuteration of that same peptide, plotted over the deuteration time. The two depicted peptides are the only peptides covering the key region within the BH3 domain of BCL-2^∆TMD^ D103Y. This illustrates the gap in our ΔΔG data in this area since we have such low peptide coverage in this region and both peptides contain the mutated D103(Y) residue, thereby making it impossible for us to compare the deuterium uptake of these peptides with the goal of evaluating structural dynamic shifts.

**Figure S4:**

**Fluorescence intensity of mCherry-BCL-2 is inconsistent with BCL-2 G101V.**

**A:** Representative western blot of HCT AKO cells after mCherry-BCL-2 over-expression for 24 hours.

**B:** Fluorescence intensity of mCherry-BCL-2 G101V protein measured relative to BCL-2 WT and empty mCherry vector in the Incucyte at transient transfection DNA amounts ranging from 40-100ng. To ensure constant DNA uptake levels during the transfection, the missing DNA amount was filled up with empty vectors up to 100ng.

**Figure S5:** Representative fit curve of the fractional SMAC-mCherry release showing EC_50_ (half-maximal effective concentration) for baby mouse kidney (BMK) BAX/BAK double knock-out (DKO) cells with addition of 20 nM recombinant BAX, 2 nM recombinant cBID, various concentrations of VEN (represented by different colors of fitting, replicates and 95 % confidence interval), and various concentrations of recombinant wild-type (WT) BCL-2^∆TMD^ and all other BCL-2^∆TMD^ mutants. The respective BCL-2^∆TMD^ mutations are indicated in the lower left corner of each graph. The fittings were performed on average SMAC-mCherry release values obtained from N ≥ 3 experiments (Nonlinear Bayesian modelling). The 95 % confidence interval of fit is shown, and colored points indicate individual replicates.
